# Enhanced crayfish optimization algorithm: Orthogonal refracted opposition-based learning for robotic arm trajectory planning

**DOI:** 10.1371/journal.pone.0318203

**Published:** 2025-02-05

**Authors:** Yuefeng Leng, Chunlai Cui, Zhichao Jiang

**Affiliations:** School of Mechanical Engineering, Liaoning Technical University, Fuxin, China; Northeastern University, CHINA

## Abstract

In high-dimensional scenarios, trajectory planning is a challenging and computationally complex optimization task that requires finding the optimal trajectory within a complex domain. Metaheuristic (MH) algorithms provide a practical approach to solving this problem. The Crayfish Optimization Algorithm (COA) is an MH algorithm inspired by the biological behavior of crayfish. However, COA has limitations, including insufficient global search capability and a tendency to converge to local optima. To address these challenges, an Enhanced Crayfish Optimization Algorithm (ECOA) is proposed for robotic arm trajectory planning. The proposed ECOA incorporates multiple novel strategies, including using a tent chaotic map for population initialization to enhance diversity and replacing the traditional step size adjustment with a nonlinear perturbation factor to improve global search capability. Furthermore, an orthogonal refracted opposition-based learning strategy enhances solution quality and search efficiency by leveraging the dominant dimensional information. Additionally, performance comparisons with eight advanced algorithms on the CEC2017 test set (30-dimensional, 50-dimensional, 100-dimensional) are conducted, and the ECOA’s effectiveness is validated through Wilcoxon rank-sum and Friedman mean rank tests. In practical robotic arm trajectory planning experiments, ECOA demonstrated superior performance, reducing costs by 15% compared to the best competing algorithm and 10% over the original COA, with significantly lower variability. This demonstrates improved solution quality, robustness, and convergence stability. The study successfully introduces novel population initialization and search strategies for improvement, as well as practical verification in solving the robotic arm path problem. The results confirm the potential of ECOA to address optimization challenges in various engineering applications.

## 1. Introduction

With the continuous development of robotic arm technology, robotic arm trajectory planning has become a hot topic in current robotics research. Trajectory planning is a crucial component of robotic arm motion control system technology, affecting the robot’s movement patterns and operational performance [1,2]. It can determine the relationship between time and space during the industrial robot’s working process, plan the robotic arm’s trajectory, and ensure the accuracy and efficiency of the predetermined tasks [3]. Optimized trajectory planning not only saves movement time but also reduces collisions and extends the robotic arm’s lifespan [4,5]. However, due to the complexity of robotic arm systems, including kinematic and dynamic constraints as well as operational environment uncertainties, traditional trajectory planning methods often fail to meet the demands for high efficiency and high precision. To address this issue, traditional algorithms such as numerical optimization and discrete search have been widely used but are often limited by computational resources or the inherent limitations of the algorithms themselves. In contrast, metaheuristic algorithms, with their global search capabilities and high adaptability to complex problems, show great potential in handling high-dimensional and nonlinear problems in robotic arm trajectory planning.

Metaheuristic (MH) algorithms, inspired by the behavior of natural organisms, iteratively optimize solutions by exploiting patterns found in nature, with the aim of achieving efficient results in a limited amount of time [6,7]. In recent years, many advanced swarm intelligence algorithms have emerged, such as Lévy Arithmetic Algorithm [8], Newton-Raphson-based optimizer [9], Walrus optimizer [10], Prism refraction search [11]. These algorithms have been applied to various fields and have achieved commendable results. Their simplicity, versatility, and ease of use make this class of algorithms applicable to a variety of domains, including but not limited to image segmentation [12], global optimization [13,14], path planning [15,16], agricultural monitoring [17], engineering design problems [18,19], forest fire detection [20], rescue operations [21], UAV path planning [22,23], rotor system [24], and cubic transmission [25]. This broad practical value underscores the importance of meta-heuristic algorithms in solving a variety of optimization challenges, especially where traditional deterministic methods are inadequate.

The "No free lunch" (NFL) theorem reminds us that there is no single algorithm that solves all problems [26], highlighting the value of algorithms adapted to particular challenges. Therefore, the MH algorithm specially developed for the path planning of robotic arms is crucial to improve efficiency [27]. Robotic arm path planning is a complex optimization task, demanding specialized approaches to ensure efficiency and high-quality solutions. In light of these requirements, we propose a tailored metaheuristic (MH) algorithm to address the computational challenges specific to robotic arm trajectory planning. This approach aligns with the NFL theorem, underscoring the need for targeted solutions to complex, real-world optimization tasks.

Crayfsh Optimization Algorithm (COA) is a novel intelligent optimization algorithm proposed by Jia Heming et al in 2023 [28]. Inspired by crayfish foraging, summer and competition behavior, the algorithm has fast search speed, strong search ability, and can effectively balance the global search and local search ability. However, despite these properties, COA also has limitations, such as slower convergence and a tendency to fall into local optimality. To date, only a few researchers have attempted to apply COA to manipulator trajectory planning. As with all optimization algorithms, striking an optimal balance between exploration and exploitation is crucial to determine the ideal path [29]. In essence, as an emerging algorithm, COA requires additional research and improvement to more effectively address the complex needs of trajectory planning for robotic arms.

In our study, to improve the convergence speed and exploration capability of the COA algorithm, we proposed an improved COA algorithm. During the algorithm initialization process, the tent chaotic map is used to enhance the algorithm’s randomness and diversity; Subsequently, a newly designed nonlinear dynamic adjustment factor is incorporated into the traditional COA exploration phase to dynamically adjust the search behavior for better solutions, thereby improving global search efficiency. Additionally, in the later iterations, a orthogonal refracted opposition-based learning strategy is integrated to optimize the solution space, enhancing the algorithm’s global search capability and solution quality. This ensures a balanced interaction between exploration and exploitation. Finally, adaptive factors and random factors are introduced into the contemporary population update strategy, significantly enhancing the overall performance of the algorithm.

The experimental results of 29 functions in the CEC2017 test set show that ECOA algorithm significantly improves the global optimization ability of the algorithm. This improvement effectively improves the convergence speed and accuracy of the algorithm. Experimental results show that ECOA performs better than CPSOGSA[30], GQPSO[31], EDOLSCA[32], WOA[6], SCA[33], CPO[34], SWO[35], and the original COA algorithm [28].

By solving the UAV three-dimensional trajectory planning problem, the high applicability of the ECOA algorithm to engineering problems was verified. Addressing the limitations of the COA algorithm, the ECOA algorithm was introduced and applied to robotic arm trajectory planning.

The main contributions of this study are as follows:

1. To address the limitations of the traditional COA, several key improvements were made: using the tent chaotic map for population initialization, incorporating a nonlinear dynamic adjustment factor, and integrating orthogonal refracted opposition-based learning strategy.
2. The enhanced ECOA’s exploration and exploitation capabilities were rigorously evaluated using the CEC 2017 benchmark test set. The experimental results validated the algorithm’s significant ability in optimization performance and effective solution space exploration.
3. The ECOA algorithm was applied to robotic arm trajectory planning to assess its practical application value, highlighting its high precision and efficiency in solving complex real-world engineering problems.

The second part introduces the related work of COA and MH algorithms in robotic arm trajectory planning. Section 3 provides an overview of the original algorithm structure and the proposed method. In Section 4, we conducted relevant experimental tests and performed an in-depth analysis of the proposed algorithm. Section 5 analyzes the application of the ECOA algorithm in UAV three-dimensional trajectory planning. Section 6 is the conclusion of the paper.

## 2. Related work

In recent years, the field of manipulator trajectory planning has attracted a lot of research interest due to the wide application of intelligent manipulators in various fields. Effective trajectory planning is essential for manipulators to accomplish tasks efficiently [36]. Faced with the inherent NP-hard complexity and the requirement of real-time reaction ability in manipulator trajectory planning, many researchers have deeply studied a series of optimization algorithms and strategies. Among them, swarm intelligence optimization algorithm has been widely used in manipulator trajectory planning because of its efficient and fast response ability [37]. This section aims to explore recent developments in the field, highlighting improved versions of COA and the utilization of various MH algorithms to solve trajectory planning problems in manipulator operations.

An environmental renewal mechanism, by simulating the survival habits of crayfish, was presented by Jia Haiming et al. in which water quality factors guided crayfish to find a better place. Moreover, the learning strategy based on ghost antagonism integrated in COA was helpful to enhance its ability to avoid local optimality [38]. Xiao Bingsong et al. used random search radius to optimize the foraging range, thus improving the operation efficiency of the algorithm [39]. Nebojsa Bacanin et al. improved COA by integrating COA algorithm and firefly algorithm, and improved the ability of the algorithm to escape from local optimization [40]. Meng Jiang et al. improved COA through Circle chaotic mapping to obtain more powerful global search capabilities [41].

In the past few years, the concept of oppositional learning has been widely used to improve the global search and local development capabilities of algorithms, many scholars have introduced Opposition-based Refraction Learning (ORL) into different optimization algorithms to improve their performance. Wen Long et al. proposed a novel refraction learning strategy based on the principle of light refraction, which assists the Whale Optimization Algorithm (WOA) in escaping from local optima [42]. Bilal H. Abed-alguni et al. employed a specific type of opposition-based learning, known as refraction learning, to enhance the Cuckoo Search (CS) algorithm’s capability of avoiding sub-optimal solutions [43]. Noor Aldeen Alawad and Bilal H. Abed-alguni introduced refraction learning combined with a triple mutation method (DJRL3M) to improve the DJaya algorithm for solving the Permutation Flow Shop Scheduling Problem (PFSSP) [44]. Therefore, optimization algorithms incorporating Opposition-based Refraction Learning (ORL) are often more capable of effectively balancing exploration and exploitation when addressing high-dimensional complex optimization problems, providing new directions and methodologies for current research.

There are a number of heuristic algorithms applied to trajectory planning problems of robotic arm operations recently. For instance, Lei Wang et al. in their study proposed TPBSO algorithm for solving problems of trajectory planning, particularly in the cases of robotic manipulators [45]. Jeong-Jung Kim et al. used particle swarm optimization (PSO) for trajectory optimization in robotic arm motion planning [46]. Gurjeet Singh et al. used different combinations of hybrid metaheuristic algorithms to address kinematic and trajectory planning problems. Kinematic parameters, including acceleration, deceleration, and speed, primarily affect the travel smoothness of the robot’s end effector along the trajectory path [47]. Pengfei Xin et al. proposed a particle swarm optimization-based algorithm for residual vibration suppression in spatial manipulator trajectory planning, achieving desirable results [48]. Xiaoman Cao proposed an improved multi-objective particle swarm optimization algorithm for trajectory planning in fruit-picking robotic arms [49]. Lunhui Zhang et al. proposed an efficient and highly stable adaptive cuckoo search (ACS) algorithm for time-optimal trajectory planning in serial robotic arms, minimizing total motion time under strict dynamic constraints [50]. H Guo et al. demonstrated a trajectory planning method for the safflower harvesting robotic arm based on the ant colony genetic algorithm. Then, the improved ant colony genetic algorithm realized the tasks of picking for the safflower harvesting robotic arm. The method obviously improved the picking efficiency in the safflower harvesting process [51].

Many researchers have applied metaheuristic algorithms to robotic arm trajectory planning and have made improvements, achieving good results. However, although COA has been applied in various fields, research on its application in the context of robotic arm trajectory planning remains limited. To achieve optimal trajectory planning, it is still necessary to conduct in-depth research on the two core mechanisms of swarm intelligence algorithms: exploration and exploitation.

In our study, an improved ECOA algorithm that integrates multiple strategies can effectively balance exploration and production processes. Experimental results show that the improved algorithm is superior to the existing algorithm, including CPSOGSA[30], GQPSO[31], EDOLSCA[32], WOA[6], SCA[33], CPO[34], SWO[35], and the original COA algorithm [28]. The improved algorithm has achieved good results in the trajectory planning of the robot arm.

## 3. The proposed methodology

This section briefly describes the behavior of the original COA and the corresponding mathematical model. In addition, this paper focuses on the proposed ECOA algorithm, including tent chaotic map, nonlinear dynamic adjustment factor and orthogonal refracted opposition-based learning strategy.

### 3.1. The original COA

The crayfish, also known as the red swamp crayfish or freshwater crayfish, is a crustacean living in freshwater. Due to its good feeding habits, rapid growth, fast migration, and strong adaptability, it has formed an absolute advantage in the ecological environment. Temperature changes often lead to changes in crayfish behavior. It is when the crayfish finds it too hot that it chooses to enter burrows to avoid damage from the heat; when the temperature is suitable, it chooses to crawl out of the burrows to forage. Among the crayfish, which are ectothermic animals, its behavior changes with temperature changes. It usually survives at temperatures ranging from 20°C to 35°C. The formula for the calculation of temperature is given by:

temp=rand×15+20 (1)

where *temp* represents the temperature of the crayfish’s environment, *rand* represents a number between [0,1].

#### 3.1.1. Initializing the population

In the *d*-dimensional optimization problem of COA, each crayfish is a 1×d matrix representing the solution of the problem. In a set of variables (X1, X2, X3… … Xd), the position (X) of each crayfish lies between the upper boundary (*ub*) and lower boundary (*lb*) of the search space. In each evaluation of the algorithm, an optimal solution is computed, and the solutions computed in each evaluation are compared, the optimal solution is found and stored as the optimal solution for the whole problem. The position to initialize the crayfish population is calculated using the following formula.

$$X_{i,j}=\left(ub_{j}-lb_{j}\right)\times rand+lb_{j}$$ (2)

where *X*_*i*, *j*_ represents the *i*th only crayfish in position of *j*-dimension, *ub*_*j*_ represents upper bound for the *j*-dimension, *lb*_*j*_ represents lower bound for the *j*-dimension, *rand* is 0 ~ 1 random number.

#### 3.1.2. Summer escape stage (exploration stage)

In this paper, the temperature is assumed to be 30° C as the dividing line to determine whether the current living environment is in a high temperature environment. When the temperature is greater than 30° C and it is in summer, in order to avoid the harm caused by the high temperature environment, crayfish will seek a cool and moist cave and enter the summer to avoid the influence of high temperature. The caverns are calculated as follows:

$$X_{shade}=\left(X_{G}+X_{L}\right)/2$$ (3)

where, *X*_*G*_ represents the current optimal position obtained by this evaluation number, *X*_*L*_ represents the optimal location of the current population.

The behavior of crayfish fighting for a cave is a random event. To simulate the random event of crayfish competing for the cave, we define a random number *rand*, *rand*< 0.5 indicates that no other crayfish currently compete for the cave, and the crayfish will directly enter the cave for summer. At this point, the crayfish position update calculation formula is as follows:

$$Xnew=X_{i,j}+C_{2}\times rand\times \left(X_{shade}-X_{i,j}\right)$$ (4)

where, *Xnew* represents the next generation location after the location is updated, *C*_2_ is a decline curve. *C*_2_ calculation method is as follows:

$$C_{2}=2-(t/T)$$ (5)

where *t* indicates the current number of iterations, and *T* indicates the maximum number of iterations

#### 3.1.3. Competition stage (exploitation stage)

When the temperature is greater than 30°C and rand ≥0.5, it indicates that the crayfish has other crayfish competing with it for burrows during the summer. At this point, the two crayfish will fight the cave, and crayfish Xi will adjust its position according to the position of the other crayfish Xz. The adjustment position is calculated as follows:

$$Xnew=X_{i,j}-X_{z,j}+X_{shade}$$ (6)

z=round(rand×(N−1))+1 (7)

where, *z* represents the random individual of crayfish and *N* represents the population size.

#### 3.1.4. Foraging stage (exploitation stage)

The foraging behavior of crayfish is affected by temperature, and the temperature less than or equal to 30°C is an important condition for crayfish to climb out of the cave to find food. When the temperature is less than or equal to 30°C, the crayfish will drill out of the burrow and judge the location of the food according to the optimal location obtained by this assessment, so as to find the food to complete the foraging. The position of the food is calculated as follows:

$$X_{food}=X_{G}$$ (8)

How much food crayfish eat depends on the temperature. When the temperature is between 20°C and 30°C, the crayfish has a strong foraging behavior, and the most food is found at 25°C, and the amount of food is also the largest. Thus, the food intake pattern of crayfish is approximately normal. Food intake is calculated as follows:

$$p=C_{1}\times \frac{1}{\sqrt{2\times \pi }\times \sigma }\times exp\left(-\frac{{\left(temp-\mu \right)}^{2}}{2\sigma^{2}}\right)$$ (9)

where *μ* indicates crayfish optimum temperature, σ and *C*_1_ denote crayfish feed intake under the different temperature control parameters.

The food crayfish get depends not only on the amount of food they eat, but also on the size of the food. If the food is too big, the crayfish can’t eat the food directly. Before eating food, they need to tear it apart with their claws. The size of food is calculated as follows:

$$Q=C_{3}\times rand\times \left(fitness_{i}/fitness_{food}\right)$$ (10)

where *C*_3_ indicates food factor which represents the largest food, its value is 3. *fitness*_*i*_ represents the fitness value of the ith only crayfish, *fitness*_*food*_ represents the fitness value of the location of food.

Crayfish use the value of maximum food *R* to judge the size of the food obtained and thus decide the feeding method. When *Q* > (*C*_3_+ 1)/2, the food is too big, small lobster cannot eat directly, need to use claws ripping food, eating alternately with the second and third leg. The recipe for shredding food is as follows:

$$X_{food}=X_{food}\times exp\left(-\frac{1}{Q}\right)$$ (11)

Once the food has been torn down to an easy-to-eat size, pick it up with your second and third PAWS and place it alternately in your mouth. In order to simulate the bipedal feeding process, the mathematical model of sine function and cosine function was used to simulate the alternating feeding process of crayfish. The crayfish alternate feeding formula is as follows:

$$X_{new}=X_{i,j}+X_{food}\times p\times (\cos(2\times \pi \times rand)-\sin(2\times \pi \times rand))$$ (12)

When *Q*≤(*C*_3_ + 1)/2, it indicates that the food size at this time is suitable for crayfish to feed directly, and crayfish will move directly to the food location and feed directly. The recipe for feeding crayfish directly is as follows:

$$X_{new}=\left(X_{i,j}-X_{food}\right)\times p+p\times rand\times X_{i,j}$$ (13)

### 3.2. The proposed ECOA

Considering the aforementioned analysis, we improved the COA algorithm from three perspectives:

1. Using the tent chaotic map to initialize the positions of crayfish. Leveraging its nonlinear and dynamic characteristics, this method can generate a more diverse set of initial solutions, aiding the algorithm in searching a broader solution space.
2. During the crayfish’s Summer escape stage, a nonlinear dynamic adjustment factor is designed to adaptively adjust the search step size, enhancing the exploitation capability.
3. Using orthogonal refracted opposition-based learning strategy to increase solution diversity and reduce the risk of the algorithm getting trapped in local optima.

#### 3.2.1. Improved population initialization with tent chaotic map

A discrete, high-quality initial population can accumulate rich search experience for COA, laying the foundation for heuristic algorithm intelligent search. Existing algorithms typically use pseudo-random numbers to initialize candidate solutions. Such a configuration can maximize the algorithm’s global performance. However, the strong randomness of the algorithm prevents maintaining stable objective optimization accuracy. Additionally, relying on pseudo-random number initialization can result in insufficient population traversal, leading to a decline in population diversity. To enhance exploration capabilities and elevate the level of population diversity [52], we use chaotic maps to improve the population initialization. The tent map is a chaotic system that generates mapping relations based on probability density functions, helping to expand the search range of the initial population and improve the algorithm’s global search ability [53]. The tent mapping process is as follows:

$$x_{n+1}=\left\{\begin{array}{cc} \frac{x_{n}}{\gamma } & x_{n}<\gamma \\ \frac{1-x_{n}}{1-\gamma } & x_{n}\geq \gamma \end{array}\right.$$ (14)

where, *x*_*n*_ represents current state map, *x*_*n*+1_ state for the next generation of mapping, γ for mapping parameters, in order to ensure the initial population of ergodicity, γ = 1.1. where, *x*_*n*_ represents current state map, *x*_*n*+1_ state for the next generation of mapping, γ for mapping parameters, in order to ensure the initial population of ergodicity, γ = 1.1.

**Fig 1:**
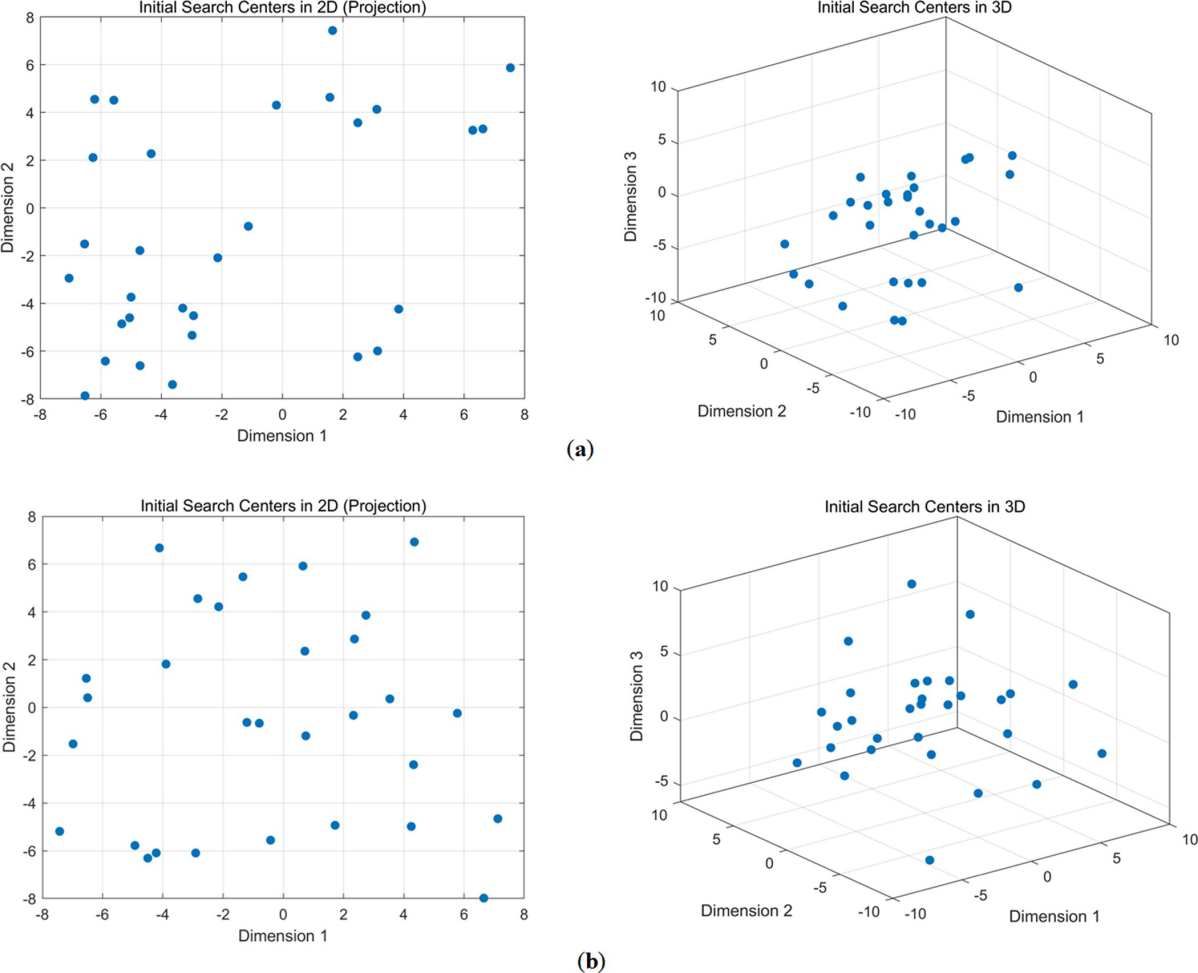
Initial population distribution. (**a**) Without tent chaotic map; (**b**) With tent chaotic map.

The scatter plot of positions initialized by the tent map is shown in Fig 1. The sequence values generated by the tent map are more evenly distributed between 0 and 1 compared to those generated by ordinary random numbers. Introducing the tent map into the initialization operation of the COA algorithm can increase population diversity and enhance the algorithm’s global search capability.

In summary, using the tent chaotic map during the COA initialization phase ensures that the initial population covers a wide solution space. This reduces the risk of premature convergence to local optima. Moreover, by diversifying the initial population, the COA algorithm can explore a broader search space, thus increasing the probability of finding the global optimal solution.

#### 3.2.2. Nonlinear dynamic adjustment factor

In the original COA algorithm, *C*_2_ is updated using Eq (5). Although this linear change can dynamically adjust the search step size to some extent, the variation of *C*_2_ is fixed, reducing solution diversity and search space coverage. The linear adjustment factor changes are fixed in each iteration, lacking randomness, which may easily lead to trapping in local optima. During the early exploration stage, the fast rate of change may lead to insufficient exploration, while in the mid-term, it may not allow for adequate leap searches. To enhance the algorithm’s global search capability, we introduced a random factor and designed a new nonlinear dynamic adjustment factor as follows:

$$C_{new}=2-\exp\left(-\frac{t^{2}}{T\times rand}\right)$$ (15)

At this point, the crawfish position update calculation is replaced by Eq (4) with:

$$Xnew=X_{i,j}+C_{new}\times rand\times \left(X_{shade}-X_{i,j}\right)$$ (16)

**Fig 2:**
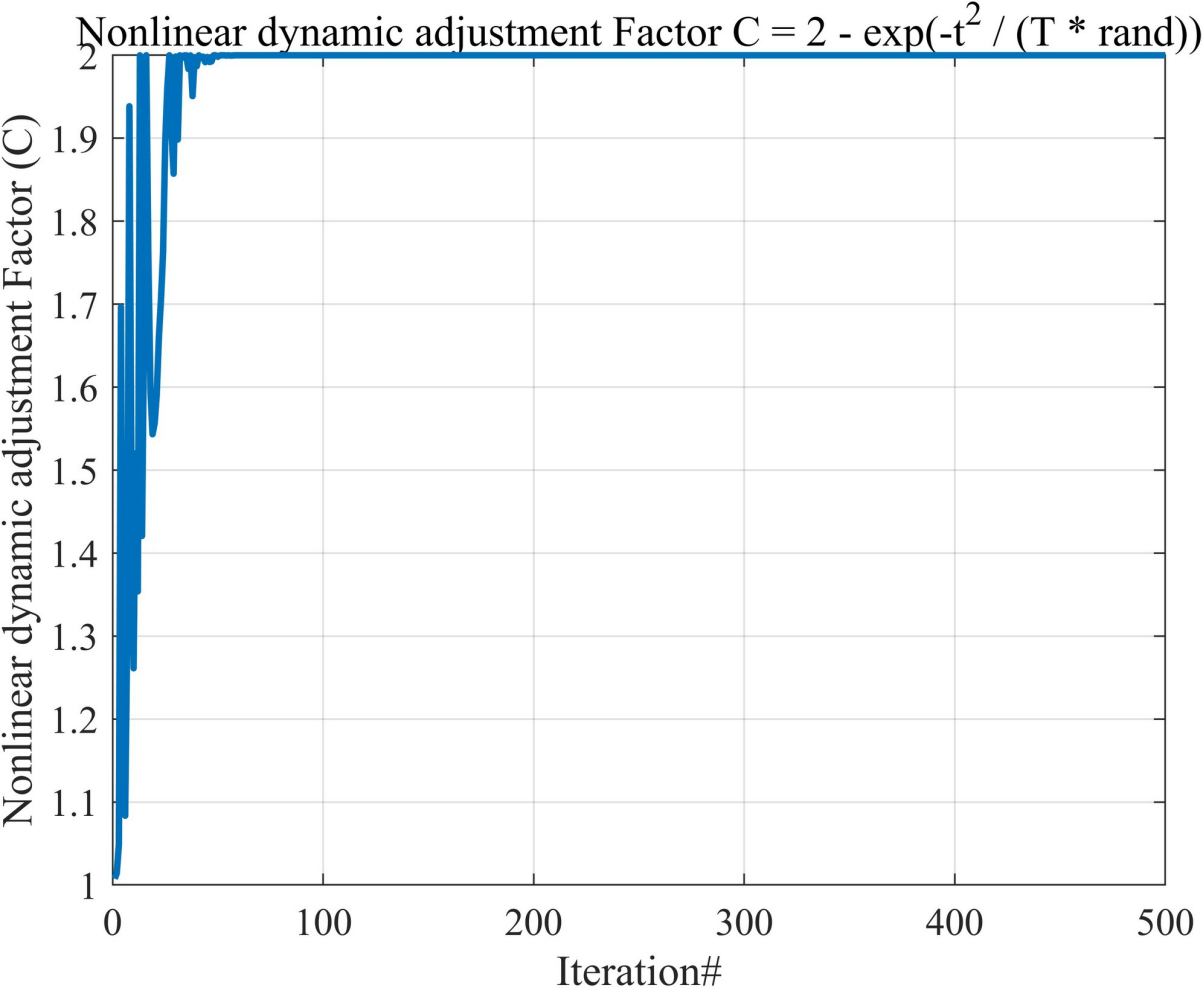
Nonlinear dynamic adjustment factor. In the mid-to-late stages, it gradually increases, which helps escape local optima and enables broader searches.

In Eq (5), *C*_2_ decreases linearly from 2 to 1. Although this linear change is simple and intuitive, the rapid decrease in the early stages may lead to a premature loss of exploration ability. Moreover, since the step size and direction of each iteration are predictable, the risk of the algorithm getting trapped in local optima increases. Eq (15) provides a new nonlinear dynamic adjustment method, making *C*_*new*_ have smaller initial values and slower changes, suitable for stable exploration. In the mid-to-late stages, it gradually increases, which helps escape local optima and enables broader searches. Its rate of change is influenced by a combination of factors and random numbers, providing high flexibility and adaptability. The iterative trend plot of *C*_*new*_ is shown in Fig 2. The random factor rand in Eq (15) introduces a certain randomness to *C*_*new*_ in each iteration, further increasing solution diversity and avoiding local optima.

#### 3.2.3. Orthogonal refracted opposition-based learning strategy

**Fig 3:**
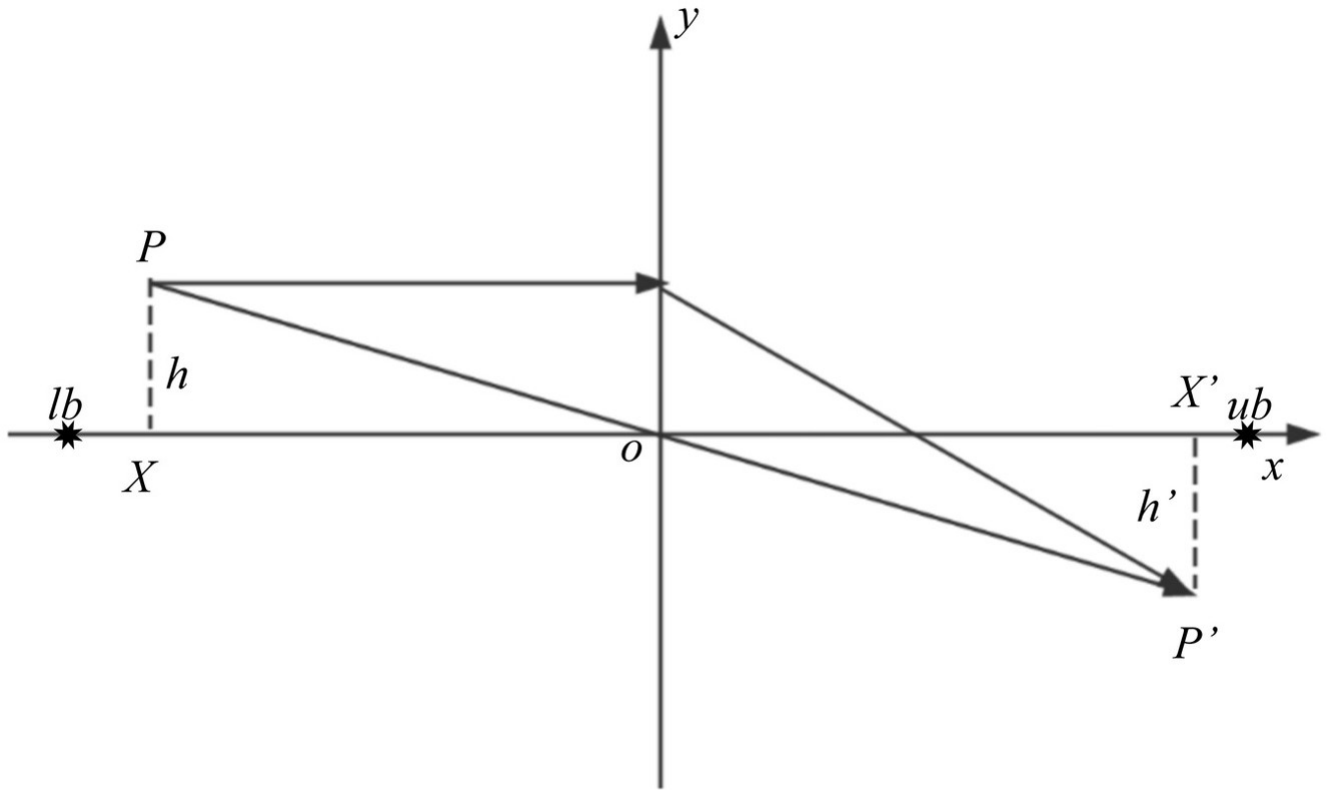
Principle of lens imaging. The optimal solution is sought by generating the reverse position according to the current individual position.

In view of the weak ability of MH algorithm to jump out of local optimum, lens imaging opposition-based learning strategy (LOBL) [54,55] is usually introduced to improve the performance of the algorithm, and the optimal solution is sought by generating the reverse position according to the current individual position. The principle of lens imaging is shown in Fig 3.

As shown in Fig 3, suppose that there exists an individual *P* in the spatial extent of the interval [*lb*, *ub*] with height h and projection *X* on the *x*-axis. By imaging with a convex lens placed at point *o* (which is the midpoint of [*lb*, *ub*]), *P*’of height *h*’ can be obtained, and its projection on the *x*-axis is *X*’. Then the imaging principle can be obtained as follows:

$$\frac{\frac{ub+lb}{2}-X}{X^{\prime }-\frac{ub+lb}{2}}=\frac{h}{h^{\prime }}$$ (17)

where, let *h*/*h*′ = *k*, and transform the formula to get:

$$X^{\prime }=\frac{ub+lb}{2}+\frac{ub+lb}{2k}-\frac{X}{k}$$ (18)

The scaling factor *k* is calculated as follows:

$$k={\left(1+{\left(t/T\right)}^{2}\right)}^{10}$$ (19)

The lens imaging reverse learning strategy explores previously uncovered areas in the solution space by reflecting and scaling solutions, thereby increasing solution diversity and reducing the risk of the algorithm falling into local optima. Additionally, in the later stages of the algorithm, when the *k* value is large, the newly generated solutions will be more concentrated around the current optimal solution. This helps the algorithm to fine-tune these solutions more precisely, accelerating convergence to the global optimum or near-global optimum solutions.

Orthogonal experimental design (OED) can find the optimal experimental combination of multi-factor and multi-level verification through a small number of tests [56]. For example, for an experiment with 2 levels and 7 factors, if a full factorial test is used to identify the optimal combination, 2^7^ = 128 tests are required. If the orthogonal experimental design is used, based on the orthogonal table *L*_8_ (2^7^) as shown in Eq (20), the optimal or near-optimal combination can be found with only 8 tests, significantly improving the experimental efficiency. However, due to the characteristics of the orthogonal experimental design, it cannot guarantee that the solutions in the orthogonal table contain the true optimal solution of the experiment [56]. Therefore, when using orthogonal tables, it is generally necessary to perform factor analysis to identify the theoretical optimal combination, and then determine the final optimal solution by comparing it with all the combinations in the orthogonal table. Thus, for the experiment with 2 levels and 7 factors, it is necessary to first obtain 8 candidate optimal solutions based on the orthogonal table *L*_8_ (2^7^), then conduct factor analysis to identify a theoretically optimal combination, and finally evaluate the 9 combinations to determine the overall optimal solution for the experiment.


$$L_{8}\left(2^{7}\right)=\left[\begin{array}{lllllll} 1 & 1 & 1 & 1 & 1 & 1 & 1\\ 1 & 1 & 1 & 2 & 2 & 2 & 2\\ 1 & 2 & 2 & 1 & 1 & 2 & 2\\ 1 & 2 & 2 & 2 & 2 & 1 & 1\\ 2 & 1 & 2 & 1 & 2 & 1 & 2\\ 2 & 1 & 2 & 2 & 1 & 2 & 1\\ 2 & 2 & 1 & 1 & 2 & 2 & 1\\ 2 & 2 & 1 & 2 & 1 & 1 & 2 \end{array}\right]$$ (20)

In order to enhance the ability of COA algorithm to jump out of local optimum, this paper proposes a strategy called orthogonal lens opposition-based learning (OLOBL), and applies it to the leader individual to generate new candidate individuals.

OLOBL is a strategy designed by integrating OED and LOBL techniques. The optimal solution executes the OLOBL strategy, jumping to more promising search areas, thereby enhancing population diversity and reducing the probability of the algorithm falling into local optima. However, the study in reference [57] shows that for an individual, its opposite solution is only superior to the current solution in certain dimensions. To address this issue, an orthogonal reflection opposite learning strategy is designed by integrating OED and LOBL techniques, which fully explores each dimensional component of both the current and opposite solutions and combines their advantageous dimensions to generate a partial reflection opposite solution. To address this issue, an orthogonal reflection opposite learning strategy is designed by integrating OED and LOBL techniques, which fully explores each dimensional component of both the current and opposite solutions and combines their advantageous dimensions to generate a partial reflection opposite solution.

The OLOBL strategy is embedded into the COA algorithm, where the optimization problem’s dimension *D* corresponds to the factors in the orthogonal experimental design, and the individual and its reflection opposite solution represent the two levels in the orthogonal experimental design. The detailed process for constructing a partial reflection opposite solution is as follows: an orthogonal experiment with 2 levels and *D* factors is designed for the current solution and its reflection opposite solution, generating *M* partial reflection opposite solutions, where *M* is calculated according to Eq (21). Specifically, when generating partial opposite solutions based on the orthogonal table, if the element in the orthogonal table is 1, the value of the corresponding dimension in the trial solution is set to the value of the current solution; if the element is 2, the value of the corresponding dimension is set to that of the reflection opposite solution.


$$M=2^{\left[\log_{2}(D+1)\right]}$$ (21)

According to the characteristics of the orthogonal experimental design, all elements in the first row of the orthogonal table are 1, indicating that the first trial solution is identical to the original individual and thus does not require evaluation. The remaining *M*-1 trial solutions are combinations of the advantageous dimensions of the current individual and its reflection opposite individual, i.e., partial reflection opposite solutions, which need to be evaluated. When using orthogonal experimental design, it is necessary to perform factor analysis to identify a theoretically optimal combination that does not exist in the orthogonal table, which also requires evaluation. Therefore, executing the OLOBL strategy requires *M* function evaluations. During the evolutionary iterations, the OLOBL strategy is only applied to the leader, and the superior individual is selected from the leader and its orthogonal reflection opposite solutions to enter the next generation. This approach effectively enhances the global exploration ability of the algorithm, reduces the number of function evaluations, and improves the overall performance of the algorithm.

In the orthogonal reflection opposite learning strategy, a reflection opposite learning approach based on the lens imaging principle is employed to enhance exploration of the opposite solution space, significantly reducing the probability of the algorithm falling into local optima. The orthogonal experimental design is used to construct several partial opposite solutions by taking reflection opposite values in certain dimensions, thoroughly exploring and preserving the advantageous dimensional information of both the current individual and the reflection opposite individual.

#### 3.2.4. ECOA algorithm description

**Fig 4:**
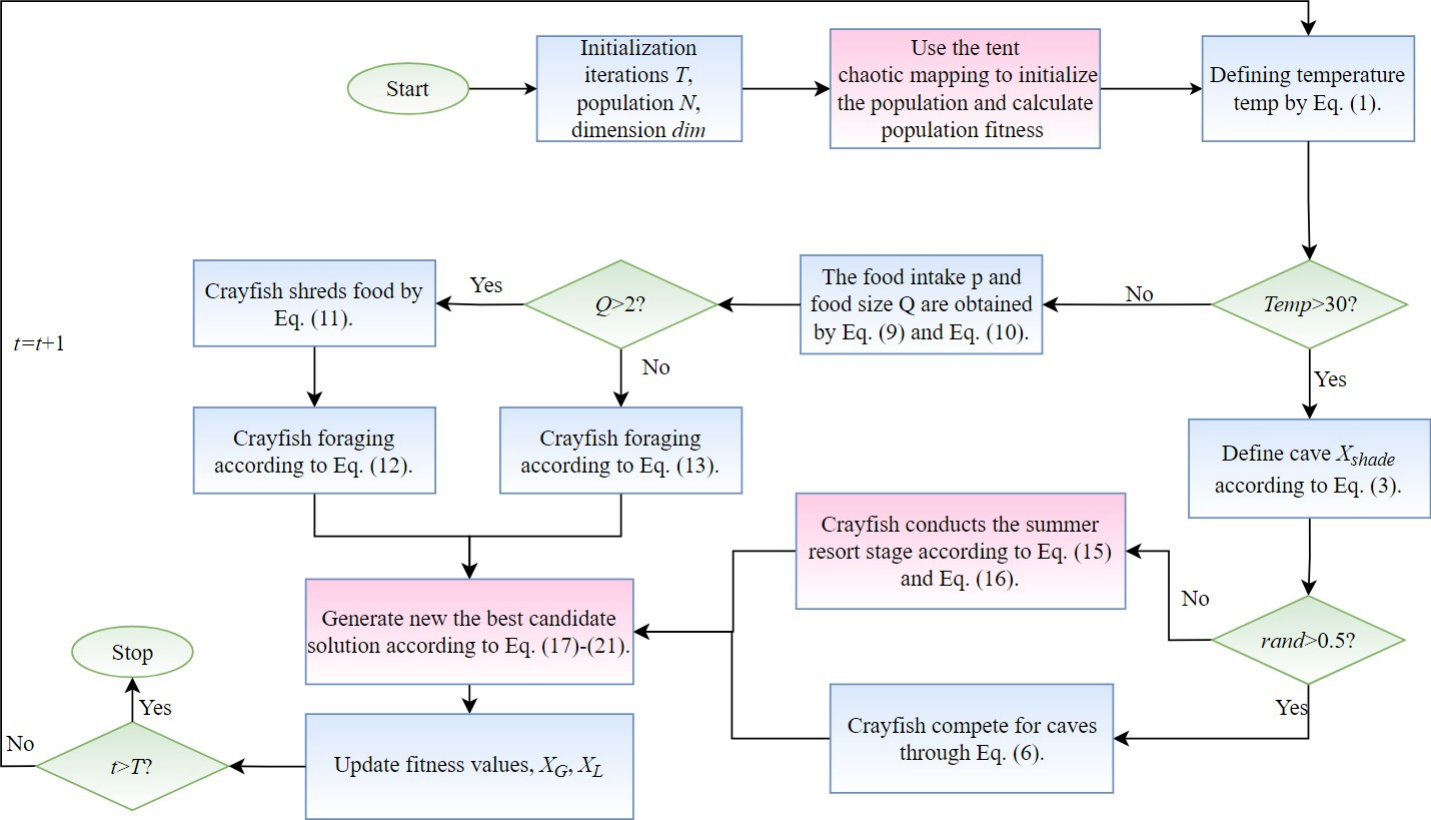
Flowchart of ECOA. The execution steps of our proposed algorithm are shown in detail.

In the improved ECOA algorithm, the tent chaotic map is used to obtain a higher quality initial solution. Additionally, during the Summer escape stage, a novel nonlinear dynamic adjustment factor is designed to replace the search step update method. This change can adaptively adjust the search step size, balancing the exploration and exploitation process of the algorithm, and further enhancing the global search capability. The OLOBL strategy is introduced in each iteration to generate and select new candidate solutions, helping the algorithm to better explore and exploit the solution space. The pseudocode for ECOA is as follows (Algorithm 1). The detailed process of ECOA is shown in Fig 4.

### Algorithm 1 ECOA

Initialization phase
Initialization iterations *T*, population *N*, dimension *dim*
**Utilize tent chaos mapping for** **population initialization**
Calculate the fitness value of the population to get *X*_*G*_, *X*_*L*_
***While*** (*t*< *T*) ***do***
 Defining temperature temp by Eq (1).
 **If** *temp*>30
  Define cave *X*_*shade*_ according to Eq (3).
  **If** *rand*<0.5
  **Crayfish conducts the summer resort stage according to Eqs (15) and (16).**
  **Else**
  Crayfish compete for caves through Eq (6).
 End
Else
 The food intake *p* and food size *Q* are obtained by Eqs (9) and (10).
 **If** *Q*>2
  Crayfish shreds food by Eq (11).
  Crayfish foraging according to Eq (12).
 **Else**
  Crayfish foraging according to Eq (13).
 End
 **Generate new the best candidate solution according to Eqs (17)-(21).**
 Calculate the fitness value of the population to get *X*_*G*_, *X*_*L*_.
End
Update fitness values, *X*_*G*_, *X*_*L*_
*t* = *t*+1
End

### 3.3. Computational complexity of ECOA

The computational complexity of the ECOA algorithm is primarily influenced by two key factors: solution initialization and the execution of core functions. These core functions encompass fitness function calculations and solution updates. The computational complexity is influenced by crucial variables, including the number of solutions (*N*), the maximum iteration limit (*T*), and the problem’s dimension (*D*). Specifically, the complexity of initializing solutions is represented as O(*N*), indicating its direct relationship with the number of solutions. As *N* increases, the computational complexity of the initialization phase also rises accordingly. The original DBO overall time complexity for the core functions of the algorithm is *O*(*T*×*N*×*D*), considering the number of iterations (*T*), the count of solutions (*N*), and the problem dimension (*D*). ECOA modifies this with Eqs (14)-(19), including enhancements to population diversity using the tent chaos mapping, a new nonlinear convergence factor is used to balance exploration and exploitation, and OLOBL strategy is introduced to obtain better solutions. The tent chaos mapping strategy, which requires computation for each individual, exhibits a complexity of O(*N*). The update from Eqs (15) and (16) is independent of population size and search dimensions, correlating only with the maximum number of iterations, resulting in a time complexity of O(*T*). Similarly, The update of Eqs (17)-(21) is independent of the population size, but only related to the maximum number of iterations and the search dimension, resulting in a time complexity of O(*T* × *D*). Furthermore, the computational complexity for both Eqs (11) and (12) is also O(*T*×*N* × *D*). Consequently, the overall time complexity of ECOA is O(ECOA)=O(N)+O(T)+O(T×D)+O(T×N×D)=O(T×N×D), consistent with the original algorithm.

## 4. Algorithm performance testing and analysis

The simulation environment of this study runs on a Windows 11 64-bit operating system, with a CPU model of AMD Ryzen 74800H, a base frequency of 2.30GHz, and equipped with 16GB RAM. The algorithms were implemented on the Matlab 2023b platform.

### 4.1. Test functions and parameter settings

To evaluate the effectiveness of the newly proposed ECOA algorithm, it was tested using the CEC2017 test function set (*dim* = 30, 50, 100). The CEC series includes a set of basic test functions that can serve not only as benchmarks for comparing the performance of various optimization algorithms but also as tools for simulating the complexity of real-world problems. This test set includes 30 CEC2017 test functions, each composed of different basic test functions. Among them, F1 to F3 are unimodal functions, F4 to F10 are multimodal functions, F11 to F20 are hybrid functions, and F21 to F30 are composite functions, the F2 function was officially removed due to its instability in high-dimensional scenarios. The search domain of the CEC2017 test function set is uniformly set to [–100, 100]^*D*^.

Comparative experiments were conducted between the ECOA algorithm and seven highly cited algorithms: CPSOGSA[30], GQPSO[31], EDOLSCA[32], WOA[6], SCA[33], CPO[34], SWO[35], and the original COA algorithm [28]. Table 1 provides a detailed summary of the parameters used in these seven different MH algorithms. The parameters for the comparative algorithms were consistent with those in the original literature. The experimental results were meticulously recorded, including the mean (denoted as "*Ave*") and standard deviation (*Std*) of each algorithm. To clearly compare performance, the best results among the nine algorithms were highlighted in bold in the table. In this study, we selected the maximum number of iterations (T) as the termination criterion because it provides a consistent and straightforward measure of algorithm performance, especially for evaluating convergence behavior under controlled computational conditions. In these tests, the population size (*N*) for each algorithm was fixed at 30, and the maximum number of iterations (*T*) was set to 500, following the settings suggested in the original COA algorithm paper and other comparison algorithms’ papers. Each experiment was independently conducted 30 times, and the system recorded the best fitness value for each trial.

**Table 1:** Parameter configurations for competing algorithms.

Algorithms	Parameter	Value
CPSOGSA	*φ*1, *φ*2	2.05, 2.05
GQPSO	*α*, *β*	0.0001, 0.0001
EDOLSCA	*w*, *Jr*	[1,15], [0.1,1]
WOA	*a*, *a*2, *b*	[0,2], [– 1,–2], 1
SCA	*α*	2
CPO	N, α, *N*min, *Tf*, *T*	120, 0.1, 80, 0.5, 2
SWO	TR, CR, Nm, N	0.3, 0.2, 20, 100
COA	*C*1, *C*3, *μ*, *σ*	0.2, 3, 25, 3
ECOA	*C*1, *C*3, *μ*, *σ*	0.2, 3, 25, 3

### 4.2. Comparative analysis of ECOA and other algorithm

The CEC2017 series of functions is a valuable tool for simulating complex real-world problems. In this study, we compared the proposed algorithm with eight other competitive algorithms: CPSOGSA[30], GQPSO[31], EDOLSCA[32], WOA[6], SCA[33], CPO[34], SWO[35], and the original COA algorithm [28]. To ensure consistency in the experimental setup, the parameters such as the number of runs, population size, test dimensions, and maximum number of iterations were kept consistent with Section 4.1.

The experiments were conducted independently 30 times, and the best fitness value for each set of trials was recorded. Tables 2–4 present the best fitness average (*Ave*) and standard deviation (*Std*) obtained from 30 repeated experiments for CPSOGSA[30], GQPSO[31], EDOLSCA[32], WOA[6], SCA[33], CPO[34], SWO[35], the original COA algorithm [28] and ECOA. The superiority of the ECOA algorithm was highlighted through comprehensive statistical analysis. The first row compiled the Wilcoxon rank-sum test results for all algorithms to reflect their performance, assessing the statistical significance of ECOA compared to other algorithms, with the significance threshold set at 5%. When the test result is *p* < 5%, it indicates a statistical difference between ECOA and the comparative algorithms; when the test result is *p* ≥ 5%, it indicates no statistical difference between ECOA and the comparative algorithms. The interpretation of these results is based on the rank-sum test: the symbols ’+’, ’-’, and ’ = ’ indicate that the optimization performance of ECOA is better than, worse than, or equal to the other algorithms, respectively. The second row provides overall ranking information derived from the final rankings by Friedman. These tables prominently display the top-ranked results, highlighting their superior performance. In each set of test functions, the algorithm with the lowest mean and standard deviation is highlighted in bold, indicating its superior performance.

**Table 2:** Test results for CEC 2017 (*dim* = 30).

ID	Metric	CPSOGSA	GQPSO	EDOLSCA	WOA	SCA	CPO	SWO	COA	ECOA
CEC2017-F1	Std	5.0380E+09	3.0172E+10	2.6324E+10	4.8411E+09	2.1624E+10	3.7154E+10	4.2135E+10	7.3252E+08	**1.1427E+08**
	Ave	3.0889E+09	1.8228E+09	7.2657E+09	1.9819E+09	4.0884E+09	7.5490E+09	9.6024E+09	1.0484E+09	**3.2693E+08**
CEC2017-F3	Std	2.4316E+05	7.5147E+04	7.2025E+04	2.5957E+05	8.5616E+04	2.0238E+05	1.4111E+05	1.1569E+05	**6.6431E+04**
	Ave	7.0064E+04	**5.4136E+03**	1.0777E+04	7.5488E+04	1.8563E+04	6.3564E+04	5.7841E+04	2.8117E+04	7.6840E+03
CEC2017-F4	Std	1.2466E+03	5.7344E+03	4.1010E+03	1.3091E+03	3.2128E+03	9.3359E+03	9.7924E+03	6.0433E+02	**5.5473E+02**
	Ave	4.7626E+02	4.2482E+02	2.1218E+03	3.3287E+02	1.1964E+03	2.5274E+03	3.9713E+03	1.0819E+02	**2.9399E+01**
CEC2017-F5	Std	7.9724E+02	8.5839E+02	7.8028E+02	8.6423E+02	8.2252E+02	9.0734E+02	9.1740E+02	7.7157E+02	**6.9808E+02**
	Ave	6.6315E+01	**1.6898E+01**	3.5882E+01	6.9099E+01	2.9065E+01	2.8129E+01	4.5049E+01	4.8446E+01	6.0989E+01
CEC2017-F6	Std	6.6747E+02	6.7462E+02	6.5586E+02	6.8138E+02	6.6423E+02	6.8595E+02	6.8758E+02	6.5518E+02	**6.5054E+02**
	Ave	1.0905E+01	**3.9253E+00**	6.1206E+00	1.1835E+01	7.0026E+00	7.7411E+00	9.3104E+00	1.3426E+01	1.2896E+01
CEC2017-F7	Std	1.7038E+03	1.2373E+03	1.2126E+03	1.3321E+03	1.2298E+03	1.4723E+03	1.4553E+03	1.2866E+03	**1.1135E+03**
	Ave	2.1686E+02	**1.7559E+01**	8.6130E+01	1.0571E+02	4.3253E+01	7.6290E+01	8.0054E+01	9.3723E+01	1.2556E+02
CEC2017-F8	Std	1.0738E+03	1.0927E+03	1.0267E+03	1.0704E+03	1.0924E+03	1.1638E+03	1.1599E+03	**9.7354E+02**	9.7819E+02
	Ave	5.9522E+01	**1.5506E+01**	3.4172E+01	4.8544E+01	2.4745E+01	3.1163E+01	3.3784E+01	3.5538E+01	3.5671E+01
CEC2017-F9	Std	8.8408E+03	8.8396E+03	**6.6050E+03**	1.1158E+04	8.2544E+03	1.4603E+04	1.3562E+04	7.9995E+03	8.0736E+03
	Ave	2.6146E+03	**7.2531E+02**	1.3600E+03	3.3620E+03	2.2092E+03	2.1384E+03	3.1998E+03	1.7635E+03	2.2830E+03
CEC2017-F10	Std	**5.3026E+03**	8.6873E+03	6.7385E+03	7.5400E+03	8.9489E+03	9.6221E+03	9.4869E+03	6.2583E+03	5.8066E+03
	Ave	4.8144E+02	**2.7925E+02**	6.9709E+02	9.2288E+02	3.1551E+02	3.8996E+02	3.3628E+02	8.4342E+02	9.5148E+02
CEC2017-F11	Std	6.1013E+03	5.1712E+03	5.4735E+03	1.0763E+04	4.0449E+03	1.2950E+04	9.4157E+03	1.7577E+03	**1.4785E+03**
	Ave	4.2474E+03	5.9749E+02	1.8040E+03	4.4469E+03	8.7697E+02	4.0426E+03	3.6149E+03	3.4082E+02	**2.3707E+02**
CEC2017-F12	Std	2.0462E+08	6.9320E+09	3.1529E+09	5.5826E+08	2.9856E+09	6.5638E+09	6.2454E+09	1.2851E+07	**4.6866E+06**
	Ave	5.9229E+08	8.5429E+08	2.3210E+09	3.0735E+08	9.5787E+08	1.6467E+09	1.9872E+09	1.1986E+07	**3.3374E+06**
CEC2017-F13	Std	6.6689E+07	4.1857E+09	1.5902E+09	1.9626E+07	1.0899E+09	2.9450E+09	3.2214E+09	2.6756E+05	**1.4497E+05**
	Ave	3.2709E+08	1.1472E+09	2.5423E+09	2.6344E+07	4.7772E+08	1.3962E+09	2.1373E+09	5.5958E+05	**2.0987E+05**
CEC2017-F14	Std	1.6036E+06	1.8983E+06	8.9813E+05	2.8441E+06	8.7003E+05	4.4436E+06	3.0639E+06	5.3016E+05	**3.0988E+05**
	Ave	2.3804E+06	**4.6467E+05**	7.9795E+05	3.3440E+06	6.6326E+05	3.2814E+06	2.3026E+06	7.8865E+05	5.3130E+05
CEC2017-F15	Std	6.1601E+04	1.4041E+08	1.4412E+07	7.3650E+06	3.7645E+07	3.0831E+08	2.5466E+08	3.2398E+04	**1.2940E+04**
	Ave	4.9772E+04	8.3289E+07	2.5415E+07	1.2505E+07	3.5506E+07	2.1677E+08	3.6739E+08	2.9105E+04	**1.0413E+04**
CEC2017-F16	Std	3.2985E+03	4.6920E+03	3.2197E+03	4.1622E+03	4.1515E+03	5.0692E+03	5.0520E+03	3.1535E+03	**3.1436E+03**
	Ave	3.4896E+02	**2.4494E+02**	3.7348E+02	7.6408E+02	2.5768E+02	4.8352E+02	4.5272E+02	3.6868E+02	4.2205E+02
CEC2017-F17	Std	2.6497E+03	3.1723E+03	2.5562E+03	2.8109E+03	2.8423E+03	3.4118E+03	3.3451E+03	2.2838E+03	**2.2792E+03**
	Ave	3.2867E+02	**1.7220E+02**	2.5575E+02	3.4893E+02	2.2294E+02	2.6787E+02	3.9139E+02	2.2855E+02	2.4060E+02
CEC2017-F18	Std	2.5833E+06	1.0895E+07	9.3725E+06	1.5972E+07	1.4004E+07	6.4166E+07	2.7172E+07	3.2265E+06	**1.3026E+06**
	Ave	2.7730E+06	2.5958E+06	1.0726E+07	1.7334E+07	7.9957E+06	4.2527E+07	2.3015E+07	3.2336E+06	**1.5551E+06**
CEC2017-F19	Std	6.0324E+06	1.2479E+08	6.6950E+07	2.6391E+07	9.7219E+07	4.3629E+08	4.3793E+08	3.5116E+04	**1.1738E+04**
	Ave	3.2778E+07	4.4821E+07	1.4522E+08	2.1478E+07	8.0229E+07	2.0373E+08	4.1251E+08	6.2530E+04	**1.1756E+04**
CEC2017-F20	Std	2.8772E+03	2.8660E+03	2.7221E+03	2.9309E+03	2.9144E+03	3.3937E+03	3.2902E+03	2.7639E+03	**2.6733E+03**
	Ave	2.6519E+02	**1.1543E+02**	1.7252E+02	2.3770E+02	1.7350E+02	1.3739E+02	1.6246E+02	2.6197E+02	1.8181E+02
CEC2017-F21	Std	2.5691E+03	2.6330E+03	2.5428E+03	2.6407E+03	2.6100E+03	2.6799E+03	2.6937E+03	2.4752E+03	**2.4500E+03**
	Ave	4.0287E+01	**1.8365E+01**	2.9129E+01	7.3834E+01	2.8970E+01	3.1563E+01	5.2369E+01	4.1761E+01	4.2639E+01
CEC2017-F22	Std	6.1333E+03	6.0158E+03	7.7130E+03	8.2550E+03	9.6205E+03	8.8920E+03	8.1603E+03	3.9131E+03	**3.0873E+03**
	Ave	1.6403E+03	**3.1561E+02**	1.2694E+03	1.8579E+03	1.7769E+03	1.7147E+03	1.6525E+03	2.3877E+03	1.7194E+03
CEC2017-F23	Std	3.0966E+03	3.3070E+03	2.9752E+03	3.1340E+03	3.0856E+03	3.3597E+03	3.3384E+03	2.8668E+03	**2.8516E+03**
	Ave	1.1849E+02	**3.4984E+01**	4.1856E+01	1.0847E+02	4.7549E+01	9.5780E+01	7.6626E+01	7.2989E+01	6.9470E+01
CEC2017-F24	Std	3.2205E+03	3.5569E+03	3.1402E+03	3.2562E+03	3.2365E+03	3.5596E+03	3.5676E+03	2.9960E+03	**2.9876E+03**
	Ave	1.1100E+02	**3.8847E+01**	5.4525E+01	1.0575E+02	4.1663E+01	1.0134E+02	1.0486E+02	7.0260E+01	4.0169E+01
CEC2017-F25	Std	3.3860E+03	3.5669E+03	3.7878E+03	3.2334E+03	3.6176E+03	4.7111E+03	4.8360E+03	2.9739E+03	**2.9497E+03**
	Ave	3.6399E+02	9.3590E+01	3.4954E+02	8.3572E+01	2.1405E+02	4.7932E+02	6.0538E+02	3.3381E+01	**3.2750E+01**
CEC2017-F26	Std	7.8157E+03	8.8717E+03	7.3494E+03	8.6358E+03	7.9438E+03	9.9720E+03	9.7886E+03	6.1314E+03	**5.9944E+03**
	Ave	8.9463E+02	**4.3319E+02**	9.2583E+02	1.5459E+03	4.4179E+02	6.9638E+02	7.9358E+02	1.3011E+03	1.5899E+03
CEC2017-F27	Std	3.3946E+03	3.9272E+03	3.4305E+03	3.4679E+03	3.5706E+03	4.1476E+03	4.0324E+03	3.3051E+03	**3.2712E+03**
	Ave	8.1370E+01	1.1760E+02	9.6321E+01	1.0670E+02	7.9177E+01	1.7444E+02	1.9239E+02	5.3015E+01	**2.4805E+01**
CEC2017-F28	Std	4.1697E+03	5.1309E+03	4.6378E+03	3.8956E+03	4.5787E+03	6.0272E+03	6.0834E+03	3.3837E+03	**3.3170E+03**
	Ave	6.7399E+02	1.0332E+02	5.8498E+02	3.0034E+02	4.6259E+02	5.6225E+02	9.2726E+02	7.2309E+01	**5.4850E+01**
CEC2017-F29	Std	4.7911E+03	5.6055E+03	4.7129E+03	5.6000E+03	5.2509E+03	6.3226E+03	6.0339E+03	4.1343E+03	**4.1219E+03**
	Ave	4.0342E+02	2.2748E+02	3.5163E+02	8.5721E+02	3.1464E+02	5.3318E+02	7.2456E+02	2.3945E+02	**2.2279E+02**
CEC2017-F30	Std	4.6016E+06	7.5089E+08	4.4282E+07	7.0491E+07	1.9432E+08	4.6015E+08	3.3740E+08	1.0196E+06	**2.6598E+05**
	Ave	7.2374E+06	2.5518E+08	3.4372E+07	6.2063E+07	4.7113E+07	2.2855E+08	1.9770E+08	1.2322E+06	**2.6935E+05**
Wilcoxon (+/ = /-)		25/0/4	28/0/1	26/0/3	29/0/0	28/0/1	29/0/0	29/0/0	15/0/14	0/29/0
Friedman Rank		3	7	4	5	6	9	8	2	**1**

**Table 3:** Test results for CEC 2017 (*dim* = 50).

ID	Metric	CPSOGSA	GQPSO	EDOLSCA	WOA	SCA	CPO	SWO	COA	ECOA
CEC2017-F1	Std	3.2681E+10	6.4955E+10	7.0530E+10	1.9705E+10	6.5341E+10	9.3195E+10	1.0058E+11	1.1495E+10	**4.3186E+09**
	Ave	1.4507E+10	**2.0899E+09**	8.1196E+09	4.6299E+09	7.9823E+09	9.3420E+09	1.2735E+10	5.1753E+09	2.7744E+09
CEC2017-F3	Std	4.4271E+05	**1.6404E+05**	1.6899E+05	2.9146E+05	2.2519E+05	6.3307E+05	3.3265E+05	3.3555E+05	2.4123E+05
	Ave	1.1449E+05	**1.0821E+04**	2.6977E+04	1.0157E+05	3.0600E+04	9.7238E+05	1.0245E+05	5.5773E+04	6.3764E+04
CEC2017-F4	Std	5.6307E+03	1.6221E+04	1.3659E+04	4.7350E+03	1.3137E+04	2.9641E+04	2.8176E+04	1.7368E+03	**1.1371E+03**
	Ave	2.5491E+03	1.1646E+03	4.2119E+03	1.2222E+03	2.8923E+03	4.5294E+03	6.2607E+03	7.1349E+02	**3.4571E+02**
CEC2017-F5	Std	1.0638E+03	1.1233E+03	1.0240E+03	1.1227E+03	1.1488E+03	1.2367E+03	1.2286E+03	9.0317E+02	**8.8968E+02**
	Ave	8.5575E+01	**1.8001E+01**	3.9992E+01	7.7893E+01	4.2281E+01	3.5662E+01	4.2185E+01	2.5903E+01	3.1245E+01
CEC2017-F6	Std	6.7979E+02	6.9203E+02	6.7446E+02	6.9424E+02	6.8417E+02	7.0424E+02	7.0320E+02	6.6571E+02	**6.6477E+02**
	Ave	1.0077E+01	**2.6793E+00**	7.5522E+00	1.1502E+01	5.2419E+00	6.7247E+00	8.0980E+00	5.6112E+00	8.4686E+00
CEC2017-F7	Std	3.1883E+03	1.7446E+03	1.7484E+03	1.9066E+03	1.8667E+03	2.1509E+03	2.0981E+03	1.7298E+03	**1.6823E+03**
	Ave	3.9972E+02	**2.9082E+01**	9.1889E+01	1.4162E+02	1.1197E+02	1.2076E+02	9.6327E+01	1.0532E+02	1.4794E+02
CEC2017-F8	Std	1.3577E+03	1.3973E+03	1.3323E+03	1.4023E+03	1.4335E+03	1.5428E+03	1.5497E+03	1.2255E+03	**1.2116E+03**
	Ave	7.8172E+01	**1.9751E+01**	5.1143E+01	6.7851E+01	3.6989E+01	5.6027E+01	4.0192E+01	3.1658E+01	5.8170E+01
CEC2017-F9	Std	**2.3490E+04**	3.2353E+04	2.4554E+04	3.8209E+04	3.2833E+04	4.8433E+04	4.5944E+04	2.9169E+04	2.8521E+04
	Ave	5.3296E+03	**1.9963E+03**	4.3703E+03	1.2252E+04	5.4811E+03	5.7607E+03	7.5586E+03	6.0408E+03	6.6039E+03
CEC2017-F10	Std	**8.6979E+03**	1.4743E+04	1.1428E+04	1.3563E+04	1.5474E+04	1.6318E+04	1.6169E+04	1.3385E+04	1.2337E+04
	Ave	8.3598E+02	5.3522E+02	8.1671E+02	1.2466E+03	**3.7358E+02**	6.0671E+02	4.8161E+02	1.1486E+03	1.6803E+03
CEC2017-F11	Std	1.9180E+04	1.4637E+04	1.2168E+04	8.7851E+03	1.2658E+04	2.9549E+04	2.5117E+04	6.2368E+03	**3.1600E+03**
	Ave	1.2225E+04	8.3342E+02	3.0932E+03	2.2217E+03	2.8736E+03	5.6295E+03	6.6482E+03	3.5552E+03	**8.1334E+02**
CEC2017-F12	Std	4.1229E+09	4.2480E+10	2.1781E+10	4.9143E+09	2.2481E+10	4.4986E+10	4.2341E+10	2.6584E+08	**9.5259E+07**
	Ave	3.2462E+09	4.0582E+09	9.0671E+09	2.8032E+09	6.2421E+09	8.3819E+09	1.0931E+10	2.9037E+08	**4.8563E+07**
CEC2017-F13	Std	8.4309E+08	2.0344E+10	7.7435E+09	5.7352E+08	6.8829E+09	2.0734E+10	1.8720E+10	1.1471E+06	**5.7443E+05**
	Ave	2.8720E+09	3.1369E+09	8.2888E+09	2.8522E+08	3.0489E+09	5.7319E+09	7.1715E+09	1.5707E+06	**4.4171E+05**
CEC2017-F14	Std	5.0556E+06	2.2156E+07	5.7462E+06	6.5220E+06	8.9625E+06	5.2300E+07	3.8773E+07	**1.2926E+06**	1.5338E+06
	Ave	5.7132E+06	7.0982E+06	7.1656E+06	6.3976E+06	5.1810E+06	2.2730E+07	2.8471E+07	**8.7324E+05**	1.2797E+06
CEC2017-F15	Std	1.7650E+06	2.8530E+09	1.5954E+09	4.3566E+07	1.0626E+09	3.7928E+09	4.0425E+09	9.2643E+04	**5.8549E+04**
	Ave	9.2781E+06	5.3724E+08	1.1167E+09	4.2081E+07	4.4670E+08	1.6768E+09	2.5708E+09	4.7407E+04	**2.5943E+04**
CEC2017-F16	Std	4.4015E+03	6.6023E+03	4.7028E+03	6.4726E+03	6.4942E+03	8.0870E+03	7.9127E+03	4.3668E+03	**4.1812E+03**
	Ave	5.6686E+02	**2.8466E+02**	6.1621E+02	1.0349E+03	4.5199E+02	6.0017E+02	8.3512E+02	6.7218E+02	7.6734E+02
CEC2017-F17	Std	4.3580E+03	5.7419E+03	4.8131E+03	4.6681E+03	5.0777E+03	7.2391E+03	7.0943E+03	3.5202E+03	**3.4296E+03**
	Ave	7.0981E+02	**2.6903E+02**	1.3009E+03	4.9439E+02	3.1558E+02	2.2415E+03	3.8282E+03	4.3509E+02	3.8419E+02
CEC2017-F18	Std	1.4698E+07	8.3056E+07	3.5629E+07	6.5713E+07	5.4506E+07	1.3705E+08	9.5815E+07	8.3102E+06	**4.9133E+06**
	Ave	2.1610E+07	2.6148E+07	4.0961E+07	3.9761E+07	2.8926E+07	4.8647E+07	5.7312E+07	9.1603E+06	**4.5332E+06**
CEC2017-F19	Std	6.6827E+05	1.4003E+09	1.2606E+09	1.9973E+07	6.7665E+08	1.4769E+09	1.6039E+09	3.7838E+05	**1.0572E+05**
	Ave	1.0454E+06	3.1002E+08	1.2548E+09	1.7151E+07	2.8346E+08	5.2591E+08	9.3705E+08	2.5291E+05	**1.2279E+05**
CEC2017-F20	Std	3.8258E+03	4.1025E+03	**3.6145E+03**	4.0591E+03	4.2749E+03	4.9086E+03	4.6614E+03	3.7707E+03	3.7335E+03
	Ave	3.2049E+02	**1.4792E+02**	3.5400E+02	2.9145E+02	2.2208E+02	2.5766E+02	3.0454E+02	1.5967E+02	2.5618E+02
CEC2017-F21	Std	2.9198E+03	2.9742E+03	2.8436E+03	3.0521E+03	2.9697E+03	3.1009E+03	3.0710E+03	2.7129E+03	**2.6705E+03**
	Ave	9.2643E+01	**2.4471E+01**	5.7618E+01	7.9042E+01	3.3078E+01	4.4293E+01	7.4918E+01	8.5015E+01	7.0287E+01
CEC2017-F22	Std	**1.0528E+04**	1.6822E+04	1.3676E+04	1.4359E+04	1.7195E+04	1.8008E+04	1.7830E+04	1.4530E+04	1.0930E+04
	Ave	1.1487E+03	**3.8309E+02**	1.0637E+03	1.1912E+03	5.1559E+02	6.4572E+02	5.3351E+02	1.3043E+03	4.8112E+03
CEC2017-F23	Std	3.6935E+03	3.9696E+03	3.5078E+03	3.8303E+03	3.7137E+03	4.2516E+03	4.2104E+03	3.2997E+03	**3.2334E+03**
	Ave	1.8688E+02	**5.4307E+01**	1.0699E+02	1.8351E+02	1.0647E+02	1.4619E+02	1.5813E+02	1.3921E+02	1.1547E+02
CEC2017-F24	Std	3.7555E+03	4.5524E+03	3.6414E+03	3.9208E+03	3.8965E+03	4.6033E+03	4.4302E+03	**3.4438E+03**	3.4572E+03
	Ave	1.5206E+02	1.0269E+02	7.4098E+01	1.5916E+02	**7.2610E+01**	1.7722E+02	1.4598E+02	1.5585E+02	1.5572E+02
CEC2017-F25	Std	6.8235E+03	8.3639E+03	8.2968E+03	5.4153E+03	9.5280E+03	1.4516E+04	1.4545E+04	3.9263E+03	**3.4694E+03**
	Ave	1.6159E+03	2.7059E+02	1.4276E+03	6.0838E+02	1.3524E+03	1.7229E+03	2.0487E+03	4.8993E+02	**1.8990E+02**
CEC2017-F26	Std	1.4445E+04	1.4051E+04	1.2774E+04	1.4737E+04	1.3967E+04	1.7410E+04	1.7082E+04	1.2038E+04	**1.1478E+04**
	Ave	2.1945E+03	**2.5455E+02**	1.2910E+03	1.4568E+03	8.5714E+02	1.1116E+03	1.3818E+03	1.2487E+03	1.7197E+03
CEC2017-F27	Std	4.3495E+03	6.2949E+03	4.5302E+03	4.8188E+03	4.9971E+03	6.4983E+03	6.1880E+03	3.8781E+03	**3.7242E+03**
	Ave	2.8749E+02	1.8979E+02	2.9086E+02	4.8277E+02	2.8196E+02	4.0943E+02	4.7151E+02	2.2452E+02	**1.6695E+02**
CEC2017-F28	Std	8.0401E+03	8.1973E+03	7.7137E+03	6.2115E+03	9.2785E+03	1.1237E+04	1.1196E+04	4.5403E+03	**4.0402E+03**
	Ave	1.7282E+03	**2.8305E+02**	1.0123E+03	7.4661E+02	1.4296E+03	1.1921E+03	1.3156E+03	3.6907E+02	3.1339E+02
CEC2017-F29	Std	7.2978E+03	1.5237E+04	7.3572E+03	9.2432E+03	8.7591E+03	1.7787E+04	1.5645E+04	5.6680E+03	**5.2824E+03**
	Ave	1.2553E+03	2.2376E+03	1.5492E+03	1.3700E+03	1.0094E+03	8.2234E+03	5.3390E+03	5.1606E+02	**4.9959E+02**
CEC2017-F30	Std	1.3719E+08	2.5991E+09	1.6270E+09	3.2674E+08	1.2867E+09	3.3089E+09	2.9904E+09	3.6085E+07	**1.1899E+07**
	Ave	1.0260E+08	4.8499E+08	1.8957E+09	2.1157E+08	3.7668E+08	1.2943E+09	1.4244E+09	1.8133E+07	**4.5008E+06**
Wilcoxon (+/ = /-)		24/0/5	28/0/1	27/0/2	28/0/11	28/0/1	29/0/0	29/0/0	17/0/12	0/29/0
Friedman Rank		3	7	4	5	6	9	8	2	**1**

**Table 4:** Test results for CEC 2017 (*dim* = 100).

ID	Metric	CPSOGSA	GQPSO	EDOLSCA	WOA	SCA	CPO	SWO	COA	ECOA
CEC2017-F1	Std	1.8383E+11	1.9068E+11	1.9295E+11	1.0804E+11	2.1332E+11	2.6358E+11	2.5745E+11	7.0424E+10	**4.9009E+10**
	Ave	5.8559E+10	**4.1852E+09**	1.3117E+10	1.2894E+10	1.3996E+10	2.0999E+10	1.6950E+10	1.2392E+10	9.8938E+09
CEC2017-F3	Std	9.7912E+05	**3.4933E+05**	5.2107E+05	9.2511E+05	5.8034E+05	5.4405E+06	1.9100E+06	7.4204E+05	3.7055E+05
	Ave	1.7619E+05	**1.5202E+04**	1.1629E+05	1.2646E+05	7.6312E+04	9.3314E+06	4.1466E+06	1.1605E+05	4.5487E+04
CEC2017-F4	Std	4.2153E+04	5.1164E+04	3.9997E+04	2.0132E+04	5.1863E+04	8.3974E+04	8.0912E+04	9.1185E+03	**5.5541E+03**
	Ave	1.3852E+04	3.2909E+03	9.6501E+03	3.4603E+03	4.8199E+03	1.1314E+04	1.5104E+04	3.0002E+03	**1.8231E+03**
CEC2017-F5	Std	2.0207E+03	1.9699E+03	1.8014E+03	1.9487E+03	2.0495E+03	2.2045E+03	2.2009E+03	1.5384E+03	**1.5264E+03**
	Ave	1.4588E+02	**2.9225E+01**	8.2625E+01	1.0860E+02	7.4314E+01	7.2077E+01	6.7146E+01	4.4735E+01	7.4618E+01
CEC2017-F6	Std	6.9136E+02	7.0535E+02	6.9051E+02	7.0942E+02	7.0454E+02	7.1679E+02	7.1743E+02	**6.7359E+02**	6.7378E+02
	Ave	8.3222E+00	**2.0442E+00**	6.0962E+00	1.1631E+01	4.4481E+00	6.5596E+00	5.6867E+00	2.4274E+00	4.0837E+00
CEC2017-F7	Std	7.6765E+03	3.5259E+03	3.5361E+03	3.8265E+03	4.1300E+03	4.2828E+03	4.1712E+03	3.4001E+03	**3.2610E+03**
	Ave	7.8931E+02	**7.6886E+01**	1.3694E+02	1.4521E+02	2.4715E+02	1.9639E+02	1.5544E+02	1.3219E+02	2.5056E+02
CEC2017-F8	Std	2.4525E+03	2.3585E+03	2.2053E+03	2.3713E+03	2.4330E+03	2.6298E+03	2.6280E+03	2.0016E+03	**1.9869E+03**
	Ave	1.6478E+02	**2.5085E+01**	7.9241E+01	1.5708E+02	5.9131E+01	6.8234E+01	8.3409E+01	4.8475E+01	8.9742E+01
CEC2017-F9	Std	5.2007E+04	7.1950E+04	6.4353E+04	7.6113E+04	9.2414E+04	1.0559E+05	1.0145E+05	**4.7498E+04**	4.9235E+04
	Ave	5.4649E+03	**3.3082E+03**	1.1594E+04	1.7626E+04	1.2451E+04	9.9542E+03	9.4742E+03	1.0500E+04	1.2840E+04
CEC2017-F10	Std	**1.9142E+04**	3.2217E+04	2.7468E+04	2.9587E+04	3.3000E+04	3.4333E+04	3.3914E+04	2.3583E+04	2.3393E+04
	Ave	1.5580E+03	**5.0615E+02**	1.8823E+03	1.3680E+03	7.3583E+02	6.9332E+02	7.7299E+02	3.4407E+03	3.2729E+03
CEC2017-F11	Std	3.3428E+05	1.6298E+05	**1.2294E+05**	3.0320E+05	1.7350E+05	3.6077E+05	2.6535E+05	2.9639E+05	2.2953E+05
	Ave	8.4320E+04	**1.7017E+04**	2.3015E+04	1.1769E+05	3.2784E+04	7.3904E+04	7.5256E+04	8.2150E+04	6.9757E+04
CEC2017-F12	Std	4.2821E+10	1.2241E+11	9.7178E+10	3.3136E+10	9.9306E+10	1.5286E+11	1.5053E+11	1.4866E+10	**5.5926E+09**
	Ave	1.7183E+10	5.2153E+09	1.5044E+10	8.7061E+09	1.3219E+10	1.9625E+10	2.5593E+10	7.8628E+09	**3.4347E+09**
CEC2017-F13	Std	3.5396E+09	2.7670E+10	2.3053E+10	2.6088E+09	1.7486E+10	3.1405E+10	3.1383E+10	8.1110E+08	**3.4080E+07**
	Ave	2.3211E+09	1.1609E+09	6.0179E+09	9.6414E+08	3.0945E+09	4.7979E+09	6.3716E+09	1.5051E+09	**3.3510E+07**
CEC2017-F14	Std	3.0970E+07	3.0335E+07	2.0434E+07	1.9698E+07	6.4094E+07	1.2051E+08	9.4438E+07	9.9549E+06	**6.6059E+06**
	Ave	2.1574E+07	4.4924E+06	1.1885E+07	6.0368E+06	2.5532E+07	5.3707E+07	4.4330E+07	5.2028E+06	**3.1973E+06**
CEC2017-F15	Std	1.2118E+09	1.2837E+10	8.7813E+09	4.3710E+08	6.5977E+09	1.3354E+10	1.3350E+10	1.6823E+07	**1.3941E+06**
	Ave	2.1794E+09	1.2708E+09	4.2999E+09	1.7614E+08	2.0723E+09	3.3884E+09	4.1754E+09	3.8628E+07	**2.2155E+06**
CEC2017-F16	Std	9.6767E+03	1.6986E+04	1.2480E+04	1.6005E+04	1.5059E+04	1.9521E+04	1.9157E+04	8.8968E+03	**8.4377E+03**
	Ave	1.2126E+03	**5.6800E+02**	1.9667E+03	2.1657E+03	1.0416E+03	1.1918E+03	2.4640E+03	1.6148E+03	1.5959E+03
CEC2017-F17	Std	4.1571E+04	2.2523E+05	1.9814E+05	2.9162E+04	7.2850E+04	1.1936E+06	1.3552E+06	7.0518E+03	**6.5665E+03**
	Ave	9.6153E+04	8.3074E+04	3.5560E+05	2.2180E+04	5.7310E+04	1.1082E+06	1.3244E+06	1.1080E+03	**1.0319E+03**
CEC2017-F18	Std	2.7068E+07	5.2830E+07	2.6142E+07	2.1053E+07	1.2696E+08	2.3134E+08	1.5159E+08	1.1857E+07	**7.1203E+06**
	Ave	1.8218E+07	1.0219E+07	1.8198E+07	1.3530E+07	5.3613E+07	9.1491E+07	6.4901E+07	1.0713E+07	**4.5736E+06**
CEC2017-F19	Std	8.0092E+08	1.1295E+10	8.6089E+09	5.3161E+08	5.2283E+09	1.3973E+10	1.3544E+10	1.5007E+07	**6.2846E+06**
	Ave	1.9855E+09	1.0448E+09	3.2617E+09	2.4906E+08	1.0662E+09	3.1163E+09	4.8281E+09	1.1430E+07	**8.5714E+06**
CEC2017-F20	Std	**6.1397E+03**	7.6410E+03	6.6121E+03	7.3092E+03	8.0244E+03	8.8428E+03	8.6649E+03	6.9722E+03	7.0272E+03
	Ave	6.1042E+02	2.8472E+02	6.5493E+02	6.2344E+02	**2.1202E+02**	3.8099E+02	3.4331E+02	5.9245E+02	4.6649E+02
CEC2017-F21	Std	4.1342E+03	4.1547E+03	3.9275E+03	4.4118E+03	4.1863E+03	4.6023E+03	4.5373E+03	3.7144E+03	**3.6107E+03**
	Ave	1.7869E+02	**5.0503E+01**	8.2554E+01	2.2803E+02	8.7967E+01	1.0954E+02	1.5153E+02	1.9527E+02	1.7718E+02
CEC2017-F22	Std	**2.2020E+04**	3.4366E+04	3.0429E+04	3.1678E+04	3.5389E+04	3.7074E+04	3.6516E+04	3.0492E+04	2.7632E+04
	Ave	1.6541E+03	**5.9726E+02**	1.5922E+03	1.2263E+03	6.2226E+02	6.3605E+02	6.6970E+02	2.8741E+03	2.6645E+03
CEC2017-F23	Std	4.9446E+03	6.8150E+03	4.7102E+03	5.3375E+03	5.2155E+03	6.6638E+03	6.5121E+03	4.2670E+03	**4.1898E+03**
	Ave	2.0775E+02	2.1491E+02	1.4192E+02	1.9393E+02	**1.3265E+02**	3.3018E+02	4.6209E+02	1.7588E+02	2.1252E+02
CEC2017-F24	Std	6.8542E+03	9.6259E+03	6.1909E+03	6.8106E+03	7.2842E+03	1.0183E+04	1.0142E+04	5.3641E+03	**5.3037E+03**
	Ave	5.8802E+02	**2.9568E+02**	3.0438E+02	3.6611E+02	3.4866E+02	7.8672E+02	7.2200E+02	3.3871E+02	5.2099E+02
CEC2017-F25	Std	2.6844E+04	1.7329E+04	1.8190E+04	1.0969E+04	2.2392E+04	2.9669E+04	2.7408E+04	7.6502E+03	**6.6224E+03**
	Ave	6.2076E+03	**5.2306E+02**	2.6980E+03	1.0180E+03	2.9089E+03	2.8816E+03	2.7255E+03	1.0066E+03	7.5814E+02
CEC2017-F26	Std	4.0626E+04	3.7516E+04	3.5982E+04	3.8243E+04	4.0782E+04	4.9711E+04	5.2074E+04	3.1480E+04	**2.7795E+04**
	Ave	4.7597E+03	**8.6214E+02**	2.6566E+03	3.1076E+03	2.7566E+03	3.3173E+03	5.4100E+03	3.6681E+03	4.6562E+03
CEC2017-F27	Std	5.6126E+03	1.0959E+04	6.6423E+03	6.2927E+03	8.8283E+03	1.1686E+04	1.1207E+04	4.5495E+03	**4.3317E+03**
	Ave	6.7259E+02	7.0444E+02	5.1939E+02	7.6633E+02	6.5487E+02	9.6362E+02	9.7517E+02	2.8432E+02	**2.6630E+02**
CEC2017-F28	Std	2.7351E+04	1.9309E+04	2.2913E+04	1.4553E+04	2.8562E+04	3.2243E+04	3.2961E+04	1.1174E+04	**9.1137E+03**
	Ave	3.9230E+03	**5.5523E+02**	2.5052E+03	1.2645E+03	3.0250E+03	3.0219E+03	3.5605E+03	1.6162E+03	1.2935E+03
CEC2017-F29	Std	2.4516E+04	8.3159E+04	5.0599E+04	2.1113E+04	3.9919E+04	1.8439E+05	1.9581E+05	1.1724E+04	**9.6959E+03**
	Ave	1.1169E+04	1.9129E+04	8.6821E+04	4.3582E+03	1.5738E+04	1.1112E+05	2.4738E+05	1.4068E+03	**9.4739E+02**
CEC2017-F30	Std	2.9574E+09	2.6461E+10	1.8699E+10	2.9750E+09	1.2831E+10	2.6029E+10	2.3112E+10	1.1465E+09	**1.0546E+08**
	Ave	5.2401E+09	1.6169E+09	7.2269E+09	1.0070E+09	2.7885E+09	5.3650E+09	5.5172E+09	1.5422E+09	**1.4064E+08**
Wilcoxon (+/ = /-)		28/0/1	29/0/0	29/0/0	28/0/1	29/0/0	29/0/0	28/0/1	17/0/12	0/29/0
Friedman Rank		3	66	4	5	7	9	8	2	**1**

In the performance comparison experiments on the CEC2017 test set with eight other advanced algorithms, ECOA performed the best. In the tests with dimensions of 30, 50, and 100, ECOA achieved the most first-place rankings out of the 29 test functions, attaining the best or near-best optimization performance on all test functions, without being the worst on any test function. This excellent performance of ECOA is mainly due to a number of key improvements introduced in the algorithm.

First, ECOA uses Tent Chaos mapping to generate diverse initial populations, effectively improving global search capabilities and reducing the risk of falling into local optima. In addition, the introduction of nonlinear dynamic adjustment factors enables ECOA to adjust the step size adaptively according to different stages, balancing exploration and development, thus improving the convergence speed and efficiency. OLOBL strategy further improves the diversity and quality of understanding, and helps the algorithm to break out of the local optimal and obtain higher quality solutions.

The statistical results of the Wilcoxon rank-sum test showed that ECOA consistently outperformed the other eight advanced algorithms in the CEC2017 function suite, highlighting its superior performance and proving the robustness of ECOA. It is the comprehensive application of Tent chaotic mapping, dynamic adjustment factor and OLOBL strategy that makes ECOA exhibit excellent performance in various dimensions and problem types, ensuring its efficiency in complex solution Spaces.

**Fig 5:**
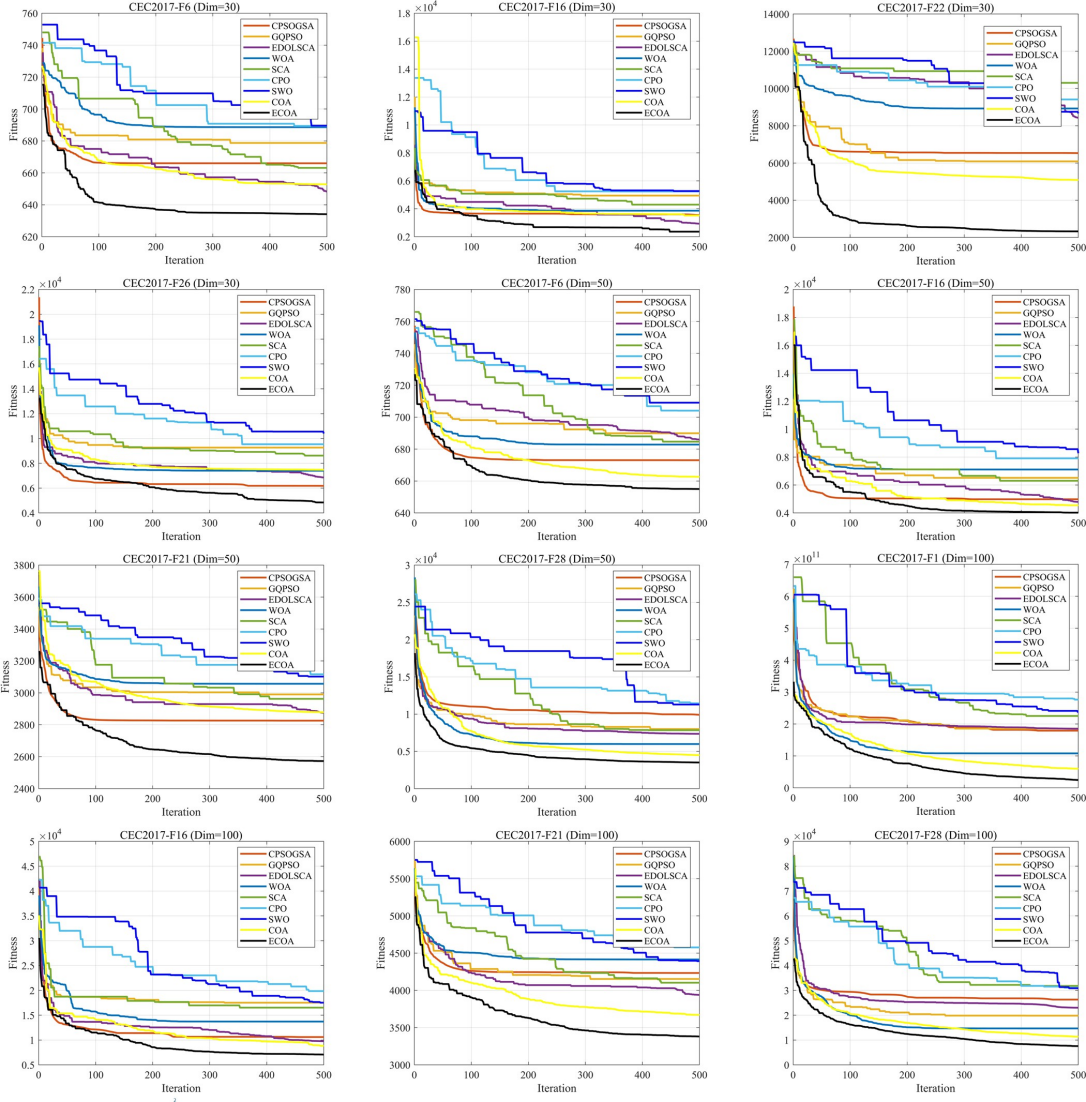
Comparison of convergence curves with different advanced algorithms. ECOA outperformed other algorithms in terms of both convergence speed and accuracy. The experimental results indicate that ECOA consistently maintained the fastest convergence speed and highest convergence accuracy.

To further analyze the convergence speed and iteration process of the aforementioned algorithms, 12 different types of test functions are selected for comparison. As shown in Fig 5, ECOA outperformed other algorithms in terms of both convergence speed and accuracy. The experimental results indicate that ECOA consistently maintained the fastest convergence speed and highest convergence accuracy, further verifying its superior performance.

In summary, ECOA is an intelligent optimization algorithm that can consistently obtain high-quality solutions. It has robust stability, fast convergence, high precision convergence, and the ability to avoid falling into local optima.

### 4.3. Ablation experiment

**Fig 6:**
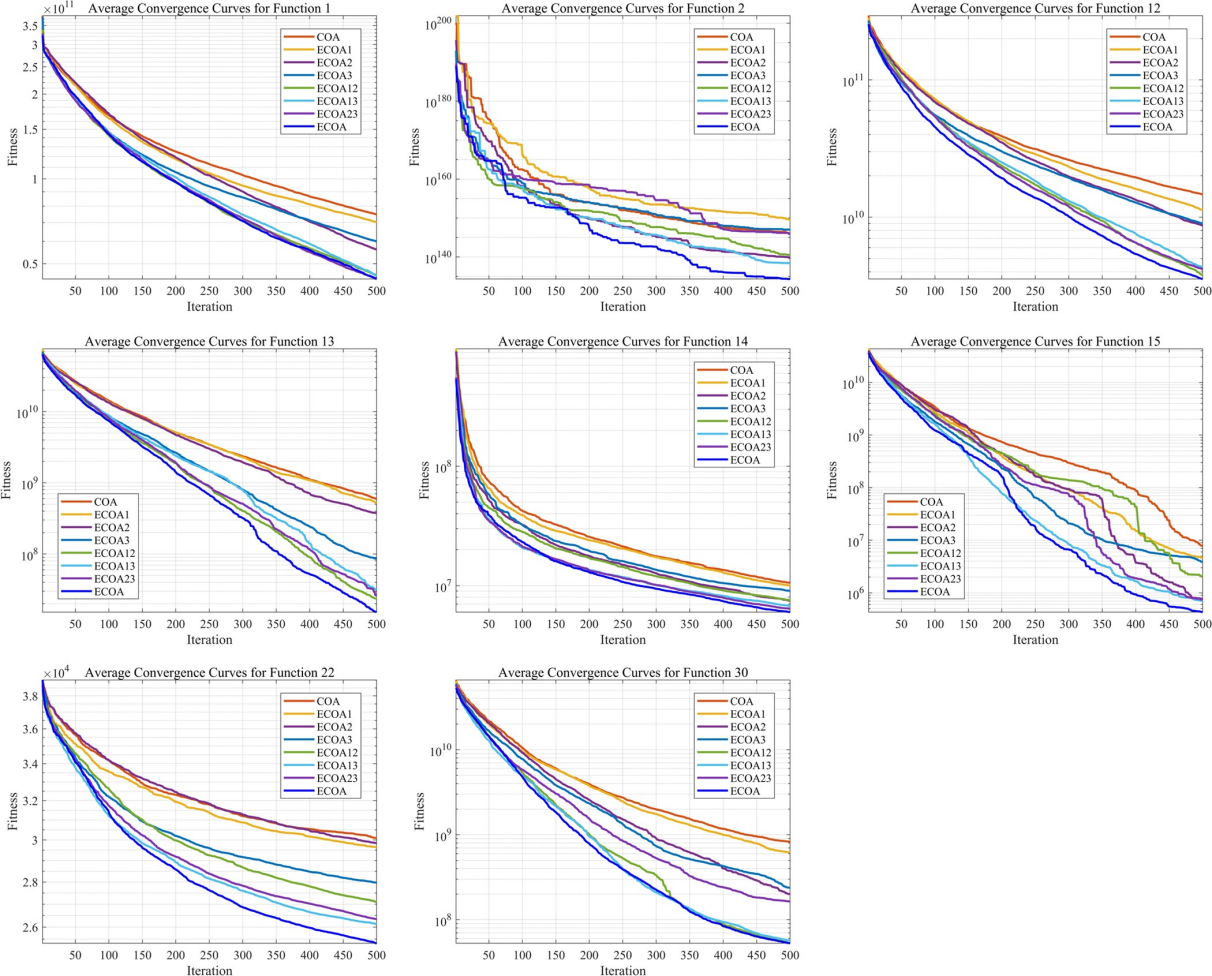
Comparison of different improvement strategies. ECOA12 (combining strategies 1 and 2), ECOA13 (combining strategies 1 and 3), and ECOA23 (combining strategies 2 and 3).

ECOA incorporates three improvement strategies: the tent chaotic map, a novel nonlinear dynamic adjustment factor, and the OLOBL strategy. To further explore the impact of these strategies on ECOA, we conducted ablation experiments in this section. Based on this, we proposed three improved algorithms: ECOA1 incorporating the tent chaotic map strategy, ECOA2 utilizing the nonlinear dynamic adjustment factor, and ECOA3 implementing the OLOBL strategy. In order to explore the interaction between strategies, we also conduct the combination of strategies. Specifically, we tested the combinations of two strategies, resulting in three additional variants: ECOA12 (combining strategies 1 and 2), ECOA13 (combining strategies 1 and 3), and ECOA23 (combining strategies 2 and 3). As shown in the experimental results in Fig 6, the 3 strategies have varying effects on COA’s performance, with ECOA demonstrating the most significant improvements.

The ablation experiments were conducted using the CEC 2017 benchmark suite (Dim = 100). When handling unimodal and multimodal functions, the results of ECOA2 and ECOA3 are relatively consistent, with both showing more noticeable improvements to COA compared to ECOA1. However, when dealing with more complex hybrid modal functions, ECOA3 demonstrates more significant enhancement effects compared to ECOA2, while the ECOA algorithm, which integrates all three strategies, continues to exhibit the best optimization performance. It is worth noting that, compared with the improvement of single strategy, the performance improvement of ECOA is more obvious after the combination of selected strategies, especially ECOA12 and ECOA13, which can conclude the effectiveness and adaptability of OLOBL strategy. The ECOA algorithm successfully overcame COA’s issues with local optima and premature convergence, significantly improving both convergence speed and accuracy. The research findings provide valuable insights for the further development and application of COA.

## 5. ECOA algorithm practical engineering application

This section is dedicated to studying the practical applications of the ECOA algorithm, particularly its application in robotic arm trajectory planning. To evaluate the performance of the ECOA algorithm, simulation experiments were conducted on the motion trajectory of a robotic arm. The simulation results verified the algorithm’s navigation capability in complex environments, making it suitable for robotic arm trajectory planning applications.

### 5.1. Trajectory planning of robot arm model

#### 5.1.1. Length of path cost

In robotic arm trajectory planning, the cost related to path length is primarily associated with the energy consumed during task execution. A shorter path generally means less energy consumption by the robotic arm during task execution. In industrial applications, reducing energy consumption is one of the key factors in lowering operational costs. To quantify this cost, a formula is designed that accurately reflects this relationship. Path length can be obtained by calculating the distance between all consecutive points. For a path in three-dimensional space, the formula for calculating the path length cost is as follows:

$$L=\sum_{i=1}^{N-1}\sqrt{{\left(x_{i+1}-x_{i}\right)}^{2}+{\left(y_{i+1}-y_{i}\right)}^{2}+{\left(z_{i+1}-z_{i}\right)}^{2}}$$ (22)

where $\left(x_{i},y_{i},z_{i}\right),\left(x_{i+1},y_{i+1},z_{i+1}\right)$ represent two consecutive points on the path, and *N* is the total number of points on the path.

#### 5.1.2. Angle of turn cost

Frequent or sharp turns may increase wear on the manipulator joints and actuation system. Optimizing the turning Angle can prolong the service life of mechanical equipment. Then the cost function of the bending Angle is:

$$C=\sum_{i=1}^{N-1}\left(cos\left(\frac{\pi }{2}\right)-\frac{v_{i}\cdot v_{i+1}}{\left\|v_{i}\|\;\|v_{i+1}\right\|}\right)$$ (23)

$$v_{i}=\left(dx_{i},dx_{i},dx_{i}\right)$$ (24)

where ***υ***_*i*_ and ***υ***_*i*+1_ represent on the path to the continuous two points.

#### 5.1.3. Height variation cost

In complex environments, such as factories or warehouses, the robotic arm needs to move among multiple objects and obstacles. Reasonably planning height changes can prevent the robotic arm from colliding with objects in the environment. Height variation H evaluates the vertical fluctuation of the path, which can be represented by the sum of the absolute differences between the heights of all points and the average height. Thus, the cost function for height variation is:

$$H=\sum_{i=1}^{N}|z_{i}-\bar{z}|$$ (25)

where $\bar{z}$ represent average value of *z*_*i*_.

#### 5.1.4. Performance measurement function of trajectory planning of robot arm

The Trajectory planning of robot arm considers three main costs: path length, angle of turn, and height variation cost. These costs have a weight coefficient *w*_1_, *w*_2_, *w*_3_. On this basis, the objective function of trajectory planning of robot arm is transformed into the weighted sum of different cost components. The formula is designed to strike a balance between various factors to determine the most efficient operating trajectory. The objective function of trajectory planning of robot arm is defined as follows:

$$F=w_{1}\;L+w_{2}\;C+w_{3}\;H$$ (26)

where *w*_1_, *w*_2_, *w*_3_ denote the weight of each item.

### 5.2. Simulation and analysis of trajectory planning of robot arm

#### 5.2.1. Algorithm application and experimental simulation

**Fig 7:**
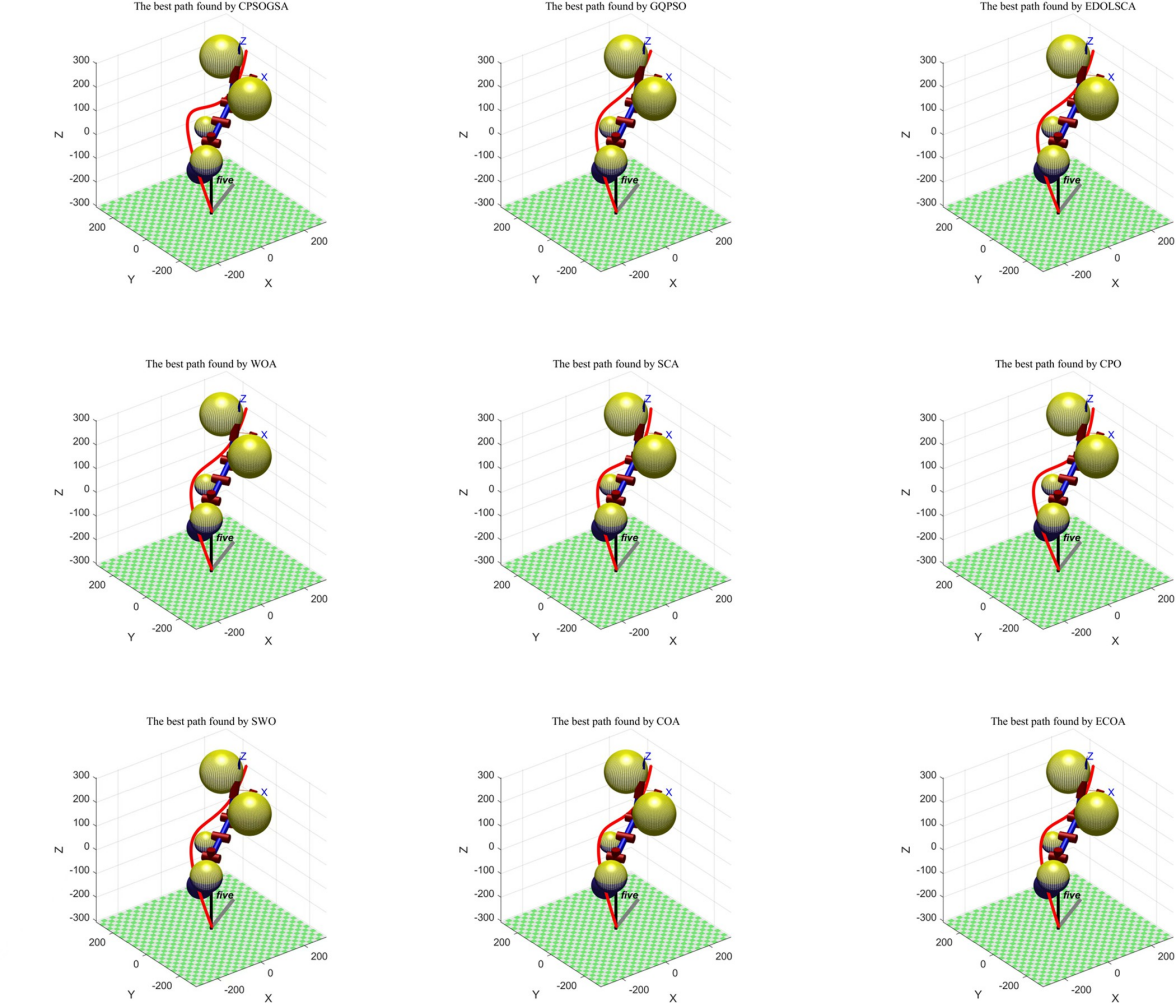
Best three-dimensional trajectory planning by each algorithm. This comprehensive simulation and analysis highlight the effectiveness of the ECOA algorithm in navigating complex environments and its potential advantages in robotic arm trajectory planning compared to other algorithms.

According to the three loss functions introduced in Section A. Trajectory planning of robot arm model, the performance measurement function of robot arm trajectory planning is defined to carry out the optimal path planning, find the path with the minimum comprehensive loss, check whether the path collide with obstacles, and abandon the path selection through obstacles, To demonstrate the performance of ECOA on the robotic arm trajectory planning problem, CPSOGSA[30], GQPSO[31], EDOLSCA[32], WOA[6], SCA[33], CPO[34], SWO[35], the original COA algorithm [28] and ECOA were also applied to the same trajectory planning problem. The parameter settings for the algorithms are as follows: population size (*N*) is 30, and the maximum number of iterations (*T*) is 200. The parameter configurations for the comparative algorithms are consistent with Section 4.1. The simulation results are shown in the figures: Fig 7 shows the three-dimensional trajectory planning results. This comprehensive simulation and analysis highlight the effectiveness of the ECOA algorithm in navigating complex environments and its potential advantages in robotic arm trajectory planning compared to other algorithms.

#### 5.2.2. Analysis of simulation result

From the analysis of the paths given by different algorithms in Fig 7, the experimental results indicate that the paths generated by the CPO and CPSOGSA algorithms tend to be longer, resulting in increased energy consumption. More importantly, their turning angles are too sharp, leading to abrupt turns, which pose significant safety hazards and increase the likelihood of robotic arm malfunctions. The trajectories of the WOA and SCA algorithms exhibit significant height variations, greatly increasing the risk of collisions. Although the routes planned by the GQPSO and SWO algorithms avoid obstacles and have smoother trajectories, they increase the path length. In contrast, the optimized ECOA algorithm successfully mitigated these issues. Overall, ECOA not only has a shorter path length but also exhibits smoother trajectories with less severe height variations, significantly reducing the risk of malfunctions.

Under the same test conditions, 30 independent simulation experiments are carried out for the nine algorithms. The comprehensive cost models of the 9 algorithms are statistically analyzed, and the relevant statistics are listed in Table 5.

**Table 5:** Statistics of trajectory planning of robot arm results.

Algorithm	Worst Cost	Best Cost	Average Cost	Midia	Standard Deviation	Wilcoxon (+/ = /-)	FriedmanValue	FriedmanRank
CPSOGSA	2.4105E+02	2.2280E+02	2.3181E+02	2.3182E+02	4.9188E+00	(+)	8.97	9
GQPSO	2.2056E+02	2.2055E+02	2.2055E+02	2.2055E+02	5.2742E-04	(+)	5.37	6
EDOLSCA	2.2055E+02	2.2055E+02	2.2055E+02	2.2055E+02	1.6850E-05	(+)	3.80	4
WOA	2.2057E+02	2.2055E+02	2.2055E+02	2.2055E+02	3.2177E-03	(-)	3.17	3
SCA	2.2055E+02	2.2055E+02	2.2055E+02	2.2055E+02	6.8729E-05	(+)	4.73	5
CPO	2.3228E+02	2.2205E+02	2.2510E+02	2.2424E+02	2.5493E+00	(+)	7.90	8
SWO	2.2410E+02	2.2116E+02	2.2244E+02	2.2218E+02	8.8934E-01	(+)	7.13	7
COA	2.2055E+02	2.2055E+02	2.2055E+02	2.2055E+02	2.4398E-13	(-)	2.08	2
ECOA	2.2055E+02	2.2055E+02	2.2055E+02	2.2055E+02	5.5700E-13	(=)	1.85	1

The analysis of the 8 sets of experimental data consistently shows that the ECOA algorithm achieved the best results across various metrics, including the optimal cost, worst cost, average cost, and median, and obtained nearly the smallest standard deviation. These results emphasize the excellent optimization performance of the ECOA algorithm, particularly in the field of robotic arm trajectory planning, where its optimization results demonstrate higher stability. The Wilcoxon rank-sum test results indicate that, except for WOA and COA, ECOA showed statistically significant performance improvements over most of the comparative algorithms. In the Friedman ranking, ECOA achieved first place, demonstrating the leading performance of the algorithm. Notably, compared to other algorithms, the ECOA algorithm starts from a significantly lower initial best fitness value, indicating its proximity to the global optimum. This characteristic significantly reduces the likelihood of the algorithm getting trapped in local optima. This characteristic significantly reduces the likelihood of the algorithm getting trapped in local optima. This stability and reliability are crucial in practical applications. In summary, the ECOA algorithm performs outstandingly in handling robotic arm trajectory planning problems.

#### 5.2.3. Discussion

The experimental results in Section 5.2 highlight the superiority of the ECOA algorithm in robotic arm trajectory planning compared to eight other algorithms. This section provides an in-depth analysis of the factors contributing to ECOA’s superior performance.

The results consistently demonstrate ECOA’s superiority over other competitive algorithms. ECOA achieved the lowest Worst Cost, Best Cost, and Average Cost, along with an exceptionally low Standard Deviation (5.57E-13), reflecting both high-quality solutions and significant robustness across multiple trials. Statistical analyses, including the Wilcoxon rank-sum test and Friedman ranking, consistently positioned ECOA as the top-performing algorithm, underscoring its superior and reliable performance.

ECOA’s remarkable performance can be attributed to several key enhancements, particularly the nonlinear dynamic adjustment factor and the orthogonal refracted opposition-based learning strategy. The nonlinear dynamic adjustment factor adapts the search behavior to the optimization phase, enabling extensive exploration during initial stages and intensifying exploitation in later stages. This adaptive mechanism is crucial for preventing premature convergence and guiding the search toward the global optimum. Additionally, the orthogonal refracted opposition-based learning strategy plays a vital role in maintaining population diversity and avoiding local optima, thereby enhancing solution quality. Together, these integrated strategies enable ECOA to outperform other algorithms by achieving shorter, smoother, and more consistent solution paths, as evidenced by the experimental data.

## 6. Conclusion

In this study, we conducted a detailed analysis of the COA algorithm, identifying its computational challenges and limitations. To address these issues, we proposed and integrated three strategic improvements: tent chaotic mapping, nonlinear perturbation factors, and orthogonal refracted opposition-based learning strategy which improves the exploration ability of the algorithm and solves the dimension degradation problem of opposition-based learning. The integration of these three strategies not only enhanced the global search capability of the COA algorithm but also improved its precision during the local optimization phase, thereby significantly accelerating the convergence speed.

Evaluations based on the CEC2017 test set in 30, 50, and 100 dimensions showed that, compared to a series of well-known algorithms, ECOA exhibited rapid convergence performance and global optimization capability. We used the Wilcoxon rank-sum test and Friedman rank-sum test to statistically verify the superiority of ECOA. The ECOA algorithm was applied to robotic arm trajectory planning and compared with eight advanced algorithms, verifying its versatility and superiority. Experimental results showed that the ECOA outperformed CPSOGSA, GQPSO, EDOLSCA, WOA, SCA, CPO, SWO, and the original COA.

Given the excellent performance demonstrated by ECOA, its application is expected to expand to a broader range of real-world challenges, such as logistics, healthcare, and energy management.

However, ECOA also has certain limitations. Our comparison with cec award-winning algorithms such as LSHADE_cnEpSin, LSHADE_SPACMA, EA4eig, and MadDE shows that ECOA does not achieve state-of-the-art performance on these challenging benchmarks. ECOA has the best applicability for manipulator trajectory planning, but does not achieve performance beyond the state-of-the-art competition algorithms on the CEC test suit. To address these challenges and further enhance the algorithm, future research will focus on integrating multiple metaheuristic strategies to better balance the efficiency of exploration and exploitation, thereby improving both efficiency and scalability.
